# Parsing digital or analog TCR performance through piconewton forces

**DOI:** 10.1126/sciadv.ado4313

**Published:** 2024-08-14

**Authors:** Aoi Akitsu, Eiji Kobayashi, Yinnian Feng, Hannah M. Stephens, Kristine N. Brazin, Daniel J. Masi, Evan H. Kirkpatrick, Robert J. Mallis, Jonathan S. Duke-Cohan, Matthew A. Booker, Vincenzo Cinella, William W. Feng, Elizabeth L. Holliday, Jonathan J. Lee, Katarzyna J. Zienkiewicz, Michael Y. Tolstorukov, Wonmuk Hwang, Matthew J. Lang, Ellis L. Reinherz

**Affiliations:** ^1^ Laboratory of Immunobiology, Dana-Farber Cancer Institute, Boston, MA 02115, USA.; ^2^ Department of Medical Oncology, Dana-Farber Cancer Institute, Boston, MA 02115, USA.; ^3^ Department of Medicine, Harvard Medical School, Boston, MA 02115, USA.; ^4^ Department of Chemical and Biomolecular Engineering, Vanderbilt University, Nashville, TN 37212, USA.; ^5^ Department of Dermatology, Harvard Medical School, Boston, MA 02115, USA.; ^6^ Department of Informatics and Analytics, Dana-Farber Cancer Institute, Boston, MA 02115, USA.; ^7^ Departments of Biomedical Engineering, Materials Science and Engineering, Physics and Astronomy, Texas A&M University, College Station, TX 77843, USA.; ^8^ Department of Molecular Physiology and Biophysics, Vanderbilt University School of Medicine, Nashville, TN 37232, USA.

## Abstract

αβ T cell receptors (TCRs) principally recognize aberrant peptides bound to major histocompatibility complex molecules (pMHCs) on unhealthy cells, amplifying specificity and sensitivity through physical load placed on the TCR-pMHC bond during immunosurveillance. To understand this mechanobiology, TCRs stimulated by abundantly and sparsely arrayed epitopes (NP_366–374_/D^b^ and PA_224–233_/D^b^, respectively) following in vivo influenza A virus infection were studied with optical tweezers. While certain NP repertoire CD8 T lymphocytes require many ligands for activation, others are digital, needing just few. Conversely, all PA TCRs perform digitally, exhibiting pronounced bond lifetime increases through sustained, energizing volleys of structural transitioning. Optimal digital performance is superior in vivo, correlating with ERK phosphorylation, CD3 loss, and activation marker up-regulation in vitro. Given neoantigen array paucity, digital TCRs are likely critical for immunotherapies.

## Teaser

Quality of ligand recognition is revealed through application of physical load on a clonal T cell receptor (TCR)–pMHC bond.

## INTRODUCTION

The vertebrate immune system is composed of both innate and adaptive cellular components that protect the host from viruses, microbes, toxins, and cancerous transformations (*1*). Innate immunity is rapid and nonspecific, while adaptive immunity is delayed but decisive, incorporating exquisite specificity and immunological memory (*2*–*4*). Adaptive humoral and cellular immunity are mediated through lymphocyte receptors, B cell receptors (BCRs) and T cell receptors (TCRs), respectively, which undergo somatic rearrangements of gene segments encoding their variable domains during lymphoid development (*5*, *6*). This process creates billions of clonotypic structures with the gamut of unique specificities required to recognize diverse pathogens. Without broad repertoire diversity, infectious agents and cancers would overwhelm the mammalian host, as evidenced by pathological sequelae observed in patients with immunodeficiency states (*7*). In contrast to BCRs, αβTCRs are exclusively membrane bound, lack affinity maturation, and manifest weak monomeric 1 to 200 μM affinities (*8*, *9*). Ligands recognized by BCRs and secreted immunoglobulins are foreign in nature, such as viral envelope proteins. On the other hand, each αβTCR recognizes a foreign peptide bound to a self–major histocompatibility complex (MHC) molecule, collectively referred to as a foreign pMHC (*5*, *10*–*14*). Foreign pMHCs are arrayed on the surface of a diseased cell or professional antigen-presenting cell (APC) at a relatively low copy number among a sea of ~100,000 diverse self-pMHCs.

Given their weak affinities, the strict specificity and sensitivity performance requirements of αβTCRs necessary for cytolytic T lymphocytes (CTLs) to eliminate abnormal cells expressing foreign pMHC were enigmatic. Recent studies solved this paradox by revealing that αβTCRs are force-responsive biomolecules, i.e., mechanosensors that, unlike antibodies, function outside of thermal equilibrium (*15*–*25*). Tensile forces applied to TCR-pMHC bonds increase their lifetimes and are referred to as catch bonds. In vivo, piconewton (pN) forces are placed on an individual αβTCR-pMHC bond through cell motions arising between a T lymphocyte and a target APC during immune surveillance (*15*, *16*). The physical load induces conformational changes in the αβTCR heterodimer, reversibly going from a compact to an elongated state, potentially delivering energy to facilitate signaling through perturbation of vicinal membrane lipids and exposure of immunoreceptor tyrosine–based activation motifs in the cytoplasmic tail of the αβTCR CD3 signaling subunits [(*20*) and references therein]. Although two studies have questioned the role of physical load on the αβTCR-pMHC bond in T cell biology (*26*, *27*), ambiguities in experimental methodologies and modeling therein cast doubt on those implications as detailed elsewhere (*14*, *28*). Multiple independent studies (*15*–*25*) and a recent review extensively referencing experimentation make apparent how mechanical force amplifies TCR mechanotransduction in T cell activation and function (*29*) emphasizing the importance of mechanobiology for cognate αβTCR recognition.

It follows that αβTCR performance without optimal mechanical load may not accurately reflect biological function in vivo, degrading ligand specificity (*17*). Notably, analysis of TCR function in vitro is routinely performed at present without such considerations (*30*, *31*). Here, we use optical tweezer (OT)–based methods to apply the equivalent of biologically relevant pN load to individual TCR-pMHC bonds, revealing differential performance of TCRs recognizing the same pMHC ligand. We discovered a force-driven “molecular resonant” state lasting minutes and involving rapid structural transitioning between contracted and extended conformations for those TCRs with the best performance. The value of physiological load application and biophysical parameterization in comparison to immunological metrics like functional avidity (*30*–*32*) or TCR sequence distance measurements per se (*33*) becomes clear from the comparison of TCRs belonging to a repertoire of T cells recognizing the same pMHC ligand. We posit that those dynamic features of an αβTCR can be linked to facile biomarkers of adaptive immune recognition performance in the future that will track with protective immunity in a clinically useful manner.

## RESULTS

### A pipeline of IAV-specific TCRs

**Fig. 1.**
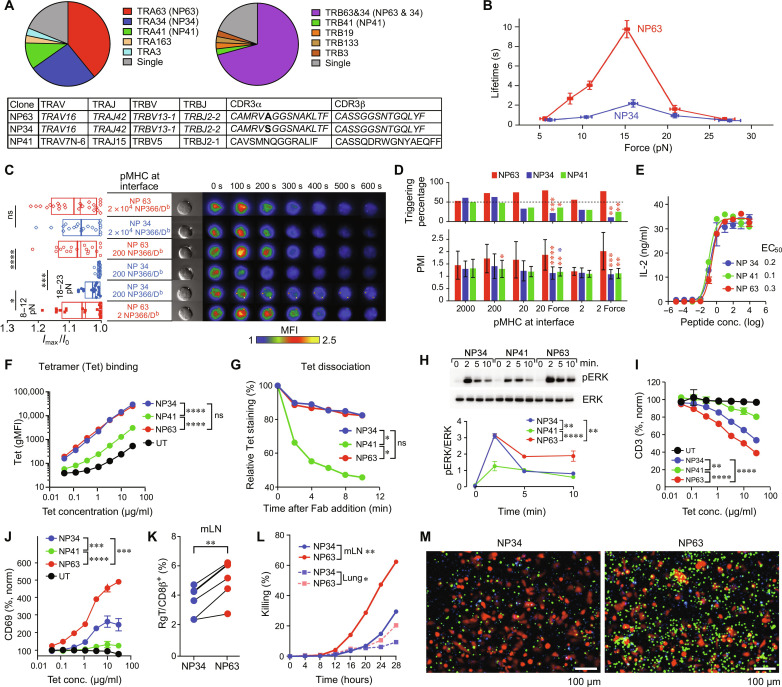
TCRs recognizing a high copy number pMHC array on IAV-infected cells function in either an analog or digital performance mode. (**A**) NP_366–374_/D^b^-specific TCR repertoire analysis. (**B**) SMSC assay measuring bond lifetime versus force (mean ± SEM). (**C**) SCAR assay measuring calcium flux magnitude and longevity. (**D**) Triggering percentage and PMI quantification using a higher-resolution dual fluorescence OT system. Red asterisks indicate comparisons of NP63 with NP34 or NP41. Blue asterisks denote comparison of NP34 versus NP41. (**E**) IL-2 production from indicated BW5147CD8αβ^+^ cells following peptide stimulation. (**F**) NP_366–374_/D^b^ tetramer (Tet) binding to TCR-transduced or untransduced (UT) BW cells following overnight treatment to trigger activation in (I) and (J). (**G**) WT tetramer dissociation. (**H**) Western blot analysis of pERK after NP_366–374_/D^b^ tetramer stimulation. (**I** and **J**) Surface CD3 loss and up-regulation of CD69 after stimulation in (F). Fluorescence intensity of CD3 (I) and CD69 (J) was normalized using the unstimulated value. (**K**) mLN Rg T cell frequency (%) of mixed RgC mice 7 days after PR8 infection. (**L**) Real-time Rg T cell killing of PR8-infected LET1 cells. (**M**) Images for mLN Rg T cells at 20 hours in (L). LET1 cells were visualized by mCherry, apoptosis by green, and Rg T cells by blue. (D) Data show mean ± SD. [(E) to (G) and (I) to (M)] Two to four independent experiments shown as means ± SDs of technical replicates [(E), (F), (I), and (J)] and of six mice (K). (H) Data are means ± SDs of three independent experiments. Small SDs are invisible [(E), (F), (H), (I), and (J)]. For statistics, ****P < 0.0001, ***P < 0.001, **P < 0.01, and *P < 0.05; ns, not significant. *P* values calculated by one-way analysis of variance (ANOVA) (C), Pearson’s chi-square test [(D), triggering percentages], Kruskal-Wallis test [(D), PMI], linear regression [(F), (I), (J), and (L)], Kolmogorov-Smirnov test (G), regression using trend line analysis models (H), or paired *t* test (K).

To identify αβTCRs directed at two immunodominant but differentially arrayed influenza A virus (IAV)-specific epitopes, nucleoprotein (NP)_366–374_/D^b^ and polymerase acidic (PA)_224–233_/D^b^, tetramers were used to concurrently isolate CD8 T lymphocytes from lung parenchyma 5 days postsecondary infection using singlecell sorting, reverse transcription polymerase chain reaction (RT-PCR) molecular cloning, and DNA sequencing (fig. S1, A and B). Of 21 MHC-bound epitopes physically identified by mass spectrometry analysis, the copy number on infected cells for NP_366–374_/D^b^ is the highest (>1000), while PA_224–233_/D^b^ is among the lowest (<10) (fig. S1C) (*34*). Figure 1A shows a representation of the TCRα (TRA) and TCRβ (TRB) clonotypes with full TRV, TRJ, and CDR3 information provided in data S1. Pie charts show the frequency of individual CDR3α (left) and CDR3β (right) clonotypes, with two or more cells colored and single-cell clonotypes in gray. The three most prevalent clonotypes with paired TRA and TRB were used for functional analysis below. NP34 and NP63 use the same Vα and Vβ gene segments, differing by only a single amino acid in Vα CDR3 (Fig. 1A, bold residue). By contrast, NP41 uses entirely different V gene segments encoding a divergent VαVβ recognition module.

### TCR recognition of dense pMHC under load

Given the near identity of the TCR sequences directed at the same pMHC, we assumed that NP63 and NP34 would yield a similar functional profile. Unexpectedly, however, clear distinctions emerged. To rule out differences in TCR copy number, adhesion molecules and/or signaling pathways leading to divergent functional outcomes, each TCR was retrovirally transduced into the same parental CD8αβ^+^ TCR^−^ BW5147 recipient cell line and selected for comparable TCRαβ expression (fig. S2A). Two key OT-based assays were used to interrogate transfectants (fig. S2, B to C) where the full CD3-TCR complex is present. Single-molecule single-cell (SMSC) measurements tether pMHC molecules to a bead through a DNA rope and present the bead to a surface of a coverslip-bound T cell. As the tethered bead is pulled away, it is displaced from the trap center and is held until the TCR/pMHC bond is broken, revealing the bond lifetime for a given force. Tether formation probability as well as peak lifetime, peak force, and width of catch bonds can also be determined. In the single-cell activation requirement (SCAR) assay, a pMHC-coated bead is trapped and moved to form interfacial contact with a T cell containing a fluorescence-based reporter of intracellular calcium concentration. An extended calcium flux in these cells is used as an indicator of T cell activation. Beads of varying pMHC densities can be used to judge TCR performance quality and determine the interfacial pMHC copy number required for activation, either with or without force. The tug by the OT mimics load between a T cell and an APC (or an infected cell) exerted through their respective actomyosin machineries (*35*).

Figure 1B demonstrates catch bond profiles. Bond lifetimes for both NP63 and NP34 TCRs peaked at a similar force, but NP63 had a fivefold longer bond lifetime than that of NP34 with peak lifetimes occurring at 15 pN for 10 s and at 16 pN for 2 s, respectively. NP41 had a bond lifetime equivalent to NP34 (fig. S2D), yet its low tethering probability (fig. S2E) required measurement using a single-molecule (SM) assay illustrated subsequently, as opposed to SMSC. The bond lifetime difference between NP63 and NP34 is further reflected by their differential responsiveness in the SCAR assay (Fig. 1C) using 2, 200, or 20,000 NP_366–374_/D^b^ copy numbers at the bead-cell interface, with or without an optimal vectorial force. The calcium flux signal is indicated as the ratio of maximum fluorescence intensity (*I*_max_) to the initial fluorescence intensity (*I*_0_) of the Ca^2+^-sensitive dye where each dot in the plot represents a single-cell experiment. The rectangles span SD with mean and median values shown as thick and thin lines, respectively. Pictures to the right are representative DIC (differential interference contrast) images of a cell-bead pair in SCAR experiment, and time-lapse images of intracellular free Ca^2+^ release for representative cells. Although both TCRs induced calcium flux with similar kinetics at high bead copy number (20,000 interfacial pMHCs per bead), only NP63 could be activated by 200 pMHCs arrayed per bead without load using this assay. Even 18- to 23-pN force application did not cause many NP34 T cells to induce calcium flux in this system. In contrast, at 8 to 10 pN, NP63 was readily activated with as little as two pMHCs per bead, the lowest interfacial ligand concentration achieved in this study.

To further elucidate activation thresholds, we adapted the above SCAR assay to a microscope specifically designed for SM fluorescence detection coupled with a sensitive camera temporally gated with excitation at very low illumination levels (see the “SCAR assay” section in Materials and Methods). These conditions virtually eliminated photobleaching and extended facile monitoring of calcium activation beyond 10 min. We thereby assayed the activation profiles more thoroughly for NP63, NP34, and NP41 at 2000 and below, in particular at 20 and 2 pMHC interfacial copy numbers with and without force. While every condition revealed an activation capability, the greatest impact observed was on the percentage of activated cells (Fig. 1D and fig. S2, F and G). The average normalized fluorescence for all cells yields the predicted mean intensity (PMI). The PMI reveals a statistically greater performance by NP63. We refer to NP63 TCR responsiveness as digital since only a couple of pMHC ligands (a few bits) are required for activation. In contrast, the NP34 and NP41 TCR responsiveness is termed analog, requiring multiple pMHC ligands to stimulate the T cell in a graded manner. Measurements on the new microscope with greater signal and reduced photobleaching revealed, more precisely, that the threshold for activating in analog cells starts to fall off at 200 interfacial molecules (Fig. 1D). On the other hand, digital cells maintain activating percentages at or above 50% (dashed line), show higher average calcium signal, and rescue their signal (PMI) with respect to both intensity and triggering percentage at 20 and 2 interfacial pMHC with force (Fig. 1D).

### Immunological assay comparisons

Functional avidity measurements using cytokine production as a readout are commonly performed to assess TCR quality through examination of T cell responsiveness to APCs cultured overnight with different peptide concentrations (*32*). The assay is technically straightforward, but its interpretation is complex, given myriad cellular components involved including adhesion molecules, coreceptors, and kinases which affect cytokine secretion. As shown in Fig. 1E, where peptide concentrations of NP_366–374_ peptide presented on R8 APCs are in nanograms per milliliter and log_10_ values are shown on the *x* axis (0 = 1 ng/ml), the functional avidity of all three NP TCRs is similar. While NP_366–374_/D^b^ tetramer binding and dissociation were comparable for NP34 and NP63 (Fig. 1, F and G, respectively) by flow cytometry using geometric mean fluorescence intensity (gMFI), the binding to NP41 was the weakest and manifested the fastest dissociation rate. The wild-type (WT) NP_366–374_/D^b^ tetramer was used for this dissociation assay because NP41 does not interact with a CD8 binding site (BS) mutant MHC molecule (fig. S3A). Collectively, these findings reveal that digital versus analog performance among TCRs cannot be discerned by the commonly used metrics of functional avidity or pMHC tetramer binding or dissociation.

Nevertheless, since tetramers can mediate cross-linking of adjacent TCR ectodomains on a T cell, themselves tethered internally to the actin cytoskeleton, we reasoned that mechanical force applied following such in vitro exposure could be used to interrogate downstream activation features. We tested whether differential mechanosensing among TCRs elicits divergent signaling responses. As the binding profiles of NP_366–374_/D^b^ tetramer for NP63, NP34, and NP41 were similar at 37°C (Fig. 1F) and at 20°C (fig. S3A), T cell activation at 37°C following tetramer binding could be readily studied. Rapid phosphorylation of extracellular signal–regulated kinase (pERK) was detected within 2 min, where the greatest magnitude and persistence were observed for NP63 (Fig. 1H and data S2). The blots on the top of Fig. 1H show one of three representative results with the pERK to ERK ratio at each time point after activation depicted in the graph below. Furthermore, the same binding leads to a differentially graded CD3ε surface loss among the three TCRs (Fig. 1I) and distinctive up-regulation of the early C-type lectin activation marker CD69 (Fig. 1J and fig. S3B).

As these studies involved in vitro assays, we next determined whether in vivo activation of NP63 and NP34 T cells differed upon IAV infection. To this end, we created single or mixed retrogenic T cell (Rg-T) mice bearing each TCR expressing T cell alone or together using fluorescence-activated cell sorting (FACS) of naïve retrogenic CD8^+^CD44^−^ T cells for those adoptive transfer experiments into B6 mice followed by IAV infection, as explained later. We quantified the mediastinal lymph node (mLN) representations at day 7 postinfection. As shown, NP63 CD8^+^ T cells expanded significantly more than NP34 in the mixed retrogenic setting and revealed the greatest incorporation of 5-Ethynyl-2’-deoxyuridine (EdU), the nucleoside analog of thymidine, into DNA during the S phase of cell cycle (Fig. 1K and fig. S4, A to C). In addition, when Rg-T cells were sorted and tested for cytolytic activity against IAV-infected mCherry^+^ lung epithelial type 1 (LET1) type I pneumocytes, as monitored continuously over 28 hours ex vivo, mLN NP63 were faster and better at killing than were NP34 T cells (Fig. 1, L and M). This was also the case for lung-derived NP63 T cells (fig. S4, D and E). The efficacy of IAV infectious doses that supports T cell–mediated killing of LET1 cells roughly correlates with intracellular LET1 NP expression by FACS analysis (fig. S4F).

However, as shown in fig. S4 (G to I), Rg-T cell numbers in lung and the level of viral titer reduction at day 7 post-IAV infection were comparable for NP34 and NP63, consistent with the notion that the high copy number of the NP_366–374_/D^b^ complexes per cell allows both digital and analog TCR performance to be effective despite the superiority of NP63 in in vitro analyses (Fig. 1). Furthermore, myriad endogenous T cells responding to this immunodominant IAV epitope in vivo probably mask the superior NP63 response to NP34 in the Rg mouse system, in contrast to the ex vivo LET1 cell killing assay that selectively examines Rg T cells in isolation. Notably, differential efficacy of digital versus analog performance in vivo may be significantly more pronounced during natural human IAV respiratory droplet–mediated infection caused by a limited number of viral particles relative to the experimental murine IAV model system used here. In the latter, a large viral inoculum is administered intranasally to anesthetized animals.

### Sparse pMHC recognition under load

**Fig. 2.**
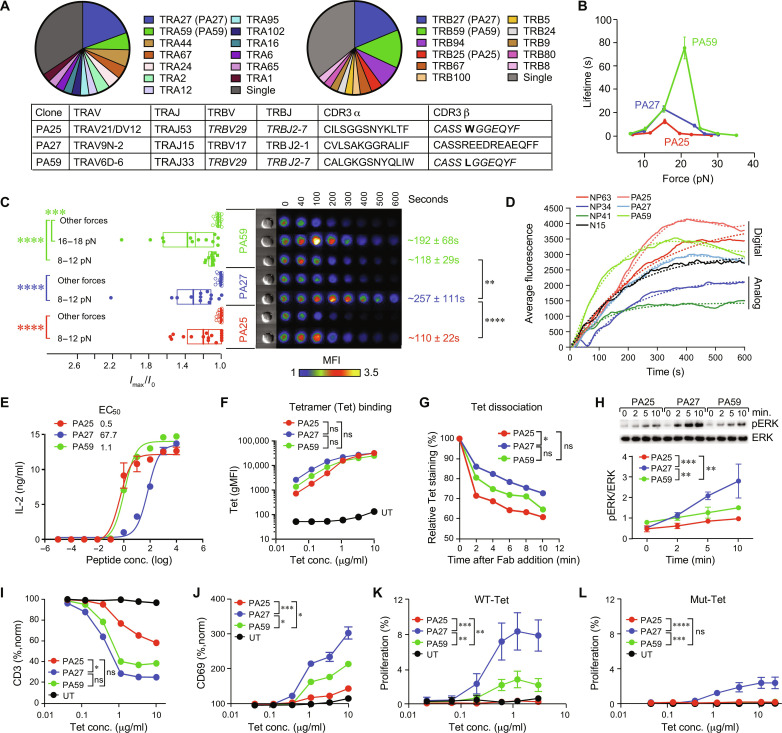
A sparse immunodominant pMHC array exclusively elicits TCRs with digital performance but distinguishable activation features. (**A**) Repertoire analysis of PA_224–233_/D^b^-specific TCRs. (**B**) SMSC measurement of bond lifetime versus force for PA25, PA27, and PA59. Data show mean ± SEM. (**C**) SCAR assay of indicated transductant with all three PA TCRs triggered by two PA_224–233_/D^b^ molecules on a bead with external force. (**D**) Average fluorescence curves of SCAR data of triggered cells (solid curves) and fits to an exponential rise (dotted curves) from the high-resolution microscope. (**E**) IL-2 assay for BW transductants after stimulation with titrated PA_224–233_ peptide. (**F**) WT PA_224–233_/D^b^ tetramer binding measured after overnight incubation. (**G**) Tetramer dissociation assay. (**H**) Western blot analysis of pERK. (**I** and **J**) Change of surface CD3 (I) and CD69 (J) expression with increasing concentration of WT tetramer as shown in (F). Indicated BW cells were incubated at 37°C overnight with tetramer, and then fluorescence intensity of CD3 (I) and CD69 (J) was measured. (**K** and **L**) Proliferation of BW transductants to WT (K) and CD8BS-mutant (L) tetramers. Measurements were obtained 30 min after addition of WT (K) or mutant (L) tetramers. Proliferation was determined by FSC-A versus SSC-A plot frequency normalized by the unstimulation value. For (E) to (G) and (I) to (L), data are representative of two to four independent experiments and mean ± SD [(E), (F), (I), and (J)] and ±SEM [(K) and (L)] of replicates shown. For (H), data are shown as mean ± SD of three independent experiments. Some error bars are invisible, given small SDs or SEMs [(E), (F), and (H) to (L)]. For all data with statistics, ****P < 0.0001, ***P < 0.001, **P < 0.01, and *P < 0.05. *P* values were calculated by one-way ANOVA [(C) and (D)], comparing slopes of linear regression [(F) and (I) to (L)], by the Kolmogorov-Smirnov test (G), and by regression using trend line analysis models (H).

Corresponding analysis of PA_224–233_/D^b^-specific TCRs (Fig. 2A and data S1) identified two common TCRs termed PA27 and PA59 and a less frequent PA25. They differ in sequence aside from PA25 and PA59 that share a Vβ gene segment and very similar CDR3β. The SMSC force versus bond lifetime analysis revealed that all three TCRs exhibited catch bond behavior, like those of the NP series (Figs. 1B and 2B). However, the PA59 maximal bond lifetime (75 s) was longer and occurred at a significantly greater force, 21 pN, relative to PA27 (23 s) and PA25 (13 s) at 15 pN, respectively. Nonetheless, all three PA TCRs manifested digital performance, being triggered by two PA_224–233_/D^b^ molecules per bead-cell interface in the SCAR assay (Fig. 2C). While PA27 and PA25 triggered well in the 8- to 12-pN range, PA59 required a higher force, i.e., 16 to 18 pN, which is consistent with the SMSC result (Fig. 2B). The duration of Ca^2+^ flux was longer for PA27, shortest for PA25, and with the greatest intensity for PA59 at high force (Fig. 2C, fig. S5A, and movies S1 to S3). The new SCAR assay sensitivity revealed that all three PAs demonstrate high triggering percentages and robust calcium signals at threshold interfacial pMHC as seen in NP63. Rescue of the PMI signal and triggering percentage are also seen at two interfacial pMHC with force (fig. S5, B and C). To directly compare relative signal levels and dynamics, individual cell calcium transients from activation through 20 and 2 interfacial molecules were pooled and averaged (Fig. 2D and data S3). Digital cells constituted the brightest intensities and exhibited slower rise time constants (NP63 = 261 s, PA25 = 259 s, PA27 = 219 s, and N15 = 185 s) compared to NP34 and NP41 which had time constants of 167 and 108 s, respectively, except for PA59 which had a time constant of 102 s (Fig. 2D). Paradoxically, the functional avidity assay indicated that PA27 was the weakest TCR based on median effective concentration (EC_50_; Fig. 2E). Of note, the interleukin-2Rα (IL-2Rα) expression (CD25) on the BW cell lines after peptide stimulation is comparable (fig. S6A). Hence, IL-2 depletion is not responsible for the discrepancy.

The above results suggest that digital PA receptors are not monolithic but rather function in distinct ways under force. Nevertheless, WT tetramer binding assays revealed no substantial differences among these TCRs (Fig. 2F and fig. S6B), while CD8BS-mutated tetramer binding was weaker only for PA25 (fig. S6B). PA27 and PA59 tetramer dissociation were similar, but both were at a slower rate than PA25 (Fig. 2G). On the other hand, WT PA_224–233_/D^b^ tetramer activation assays showed that PA27 manifested prolonged ERK-mediated activation (Fig. 2H and data S2). Persistent ERK stimulation is associated with gene activation (*36*, *37*). CD3ε down-regulation was also the largest for PA27 but not statistically distinguishable from PA59 (Fig. 2I and fig. S6C). Differential CD3 surface loss increased at higher temperature but with little change in tetramer binding, suggesting an active cellular mechanism of CD3 down-regulation likely through tetramer stimulation fostering kinase–dependent internalization (*38*, *39*) and/or dissociation of CD3 dimers from TCRαβ (fig. S6D) (*20*). PA27 also showed the greatest differential CD69 up-regulation (Fig. 2J and fig. S6C) and proliferation response to WT as well as CD8BS mutated PA_224–233_/D^b^ tetramers (Fig. 2, K and L, and fig. S6, E and F). Of note, none of the NP TCRs proliferated following CD8BS-mutated NP_366–374_/D^b^ tetramer stimulation (fig. S6G).

### SM OT analysis of digital TCRs

**Fig. 3.**
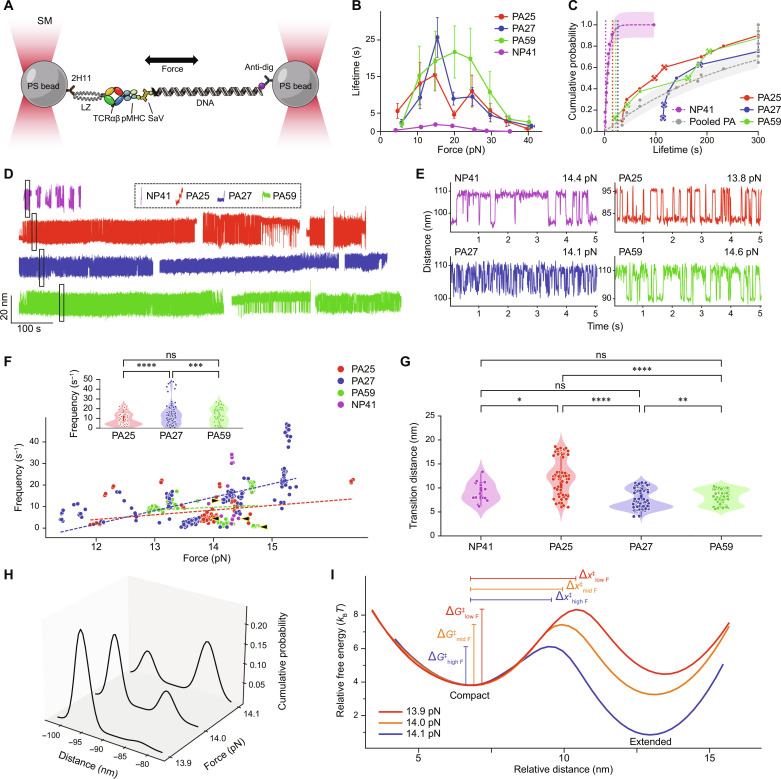
An SM dual bead OT system discriminating mechanosensing performance of digital TCRs. (**A**) SM_db_ system cartoon. (**B**) Bond lifetime versus force for PA25 (red, *n* = 175), PA27 (blue, *n* = 237), PA59 (green, *n* = 192), and NP41 (magenta, *n* = 57). Lifetimes, binned every 5 pN, are mean ± SEM. (**C**) Cumulative probability plot for continuous volleying segments. Peak lifetime from each clone in (B) is noted by a vertical dashed line. Symbols depicted by an X indicate termination by the user. PA25 (*n* = 10), PA27 (*n* = 8), PA59 (*n* = 8), and NP41 (*n* = 11). PA events were pooled (gray) and fit to the function *y* = (1 − *e*^−*x*/*t*^).The 95% confidence intervals are denoted by magenta (NP41) and gray (pooled PA) shaded areas. (**D**) Representative traces of continuous volleying segments. Traces are in the 13.8- to 14.5-pN force range and separated by a blank space. Sample traces from each catch bond curve in the same force range without reversible transitions are shown in the dotted box with similar scaling. (**E**) Magnified views of the rectangles in (D). (**F**) Hopping frequency versus force in 10-s segments. Forces on the *x* axis denote the folded state, and force decreases upon opening. Dashed lines are linear regressions. Arrowheads indicate segments in (E). Insert: Violin plots of pooled forces. (**G**) Transition distances for 10-s segments. (**H**) Example of SM position distributions versus force for PA25 in (D). Shift in population between two major states occur near the 14-pN critical force. (**I**) Free energy landscapes corresponding to (H) as given by *G* = −*k*_B_*T* ln(*P*), where *P* is the probability density. Landscapes are aligned at the compact state energy well, and barrier energies and distance to the transition state, Δ*x*, are marked. For all data with statistics, ****P < 0.0001, ***P < 0.001, **P < 0.01, and *P < 0.05. *P* values were calculated by Kruskal-Wallis tests.

We next evaluated the performance of the digital PA TCRs using SM analysis. We adapted our SM assay to a geometry where the TCR is bound to a 1.23-μm bead through the anti-leucine zipper (LZ) monoclonal antibody (mAb) 2H11, and pMHC is bound to a second 1.23-μm bead via a DNA linkage (Fig. 3A). The SM “dual-bead” assay (SM_db_) was performed by positioning the two traps to initiate tether formation followed by pulling the linkage taut to a predetermined force window. This assay was performed on a LUMICKS m-Trap microscope where microfluidic flow introduces beads to populate the traps and a measurement routine calibrates the systems.

PA27 and PA25 showed strong catch bond peaks around 15 pN, while PA59 showed a much broader distribution of lifetimes and broader peak force range. PA27 displayed the longest and narrowest peak lifetime (Fig. 3B). The wide lifetime spread, attributed to an increase in instrument response time capturing short-lived interactions for the SM_db_ assay, and variation in curve shape relative to SMSC (Fig. 2B) prompted a closer look at the lifetime distribution within each force window. A common observation was that the cumulative probability of lifetimes within each bin largely fit a double exponential model, wherein there is one time constant for quick dissociation (<2 s) and another for an extended lifetime (fig. S7A). The peak bin of ~15 pN, spanning the critical force for conformational transition, was significantly higher and demonstrated a ~45 to 50% increase in lifetime for PA27 relative to PA25 and PA59. Fit parameters converged to 29.4 ± 5.3 s and 28.2 ± 3.7 s for PA25 and PA59, respectively, compared with 42.6 ± 4.4 s for PA27 (fig. S7B). Overall, PA clones have longer bond lifetimes compared to NP41-NP_366–374_/D^b^ which exhibited a much shorter catch bond peak lifetime and more difficulty in initiating tether formation in the same SM_db_ assay (Fig. 3B).

In the SM_db_ assay, force can be incrementally altered during a measurement by slight adjustment in the trap separation. By actively maintaining tethers at or near a critical force, we were able to observe repeat reversible transitioning between extended and compact states with a corresponding extension of bond lifetime, a state we refer to as volleying. During volleying, the linkage persisted for several minutes and, in some cases, more than 5 min. To illustrate, we plot the cumulative probability of volleying lifetimes, which are much longer than the catch bond lifetimes (displayed as vertical dashes in Fig. 3C). The population of volleying segments at the 5-min lifetime mark for digital PA27, PA59, and PA25 were 25, 12.5, and 10%, respectively, although some traces were artificially ruptured by the user as noted (Fig. 3C). In contrast, although NP41 transitioned, it lasted only ~5 s on average (Fig. 3, C to E). The cumulative distribution for NP41 fit to a time constant of 6.8 ± 0.85 s. For additional comparison, we pooled lifetimes of PA-specific TCRs that either terminated naturally or survived to the 5-min mark, yielding a time constant of 264 ± 25 s which is far beyond peak catch bond lifetimes and ~40-fold longer than that of NP41 (Fig. 3C).

Long periods of sustained volleys were divided into 10-s segments for further study. Analysis of the transition frequencies versus force of these sustained volleys showed that PA25, PA27, and PA59 largely behave similarly (Fig. 3F) but that PA27 has potential to transition faster (Fig. 3F, insert) and for a slightly longer period of time (Fig. 3C). NP41 generally transitioned at a low frequency compared to the others at the same force, but all showed a spread of frequency spanning 5 to 30 Hz (Fig. 3F). Frequencies in hertz for PA27, PA25, and PA59 were 14.1 ± 10.7, 7.8 ± 6.1, and 9.5 ± 7.0 (average ± SEM), respectively (Fig. 3F, insert). The average transition distances were similar, ranging from 8 to 12 nm but with different distributions (Fig. 3G). Note the two distinct PA27 transition distances, for example. All three PA-specific clones had average critical forces for volleying in the 13.7- to 13.9-pN range, whereas NP41 volleyed on average at 14.3 pN. The critical force for volleying generally centered around ~14 pN. Even a slight pN change in force nudges the distribution of states between extended and compact (Fig. 3H). The corresponding energy landscape shows that a fractional pN increase in force shortens the distance to the transition state from compact and lowers the energy barrier to the transition (Fig. 3I).

### In vivo transcriptome of digital TCRs

**Fig. 4.**
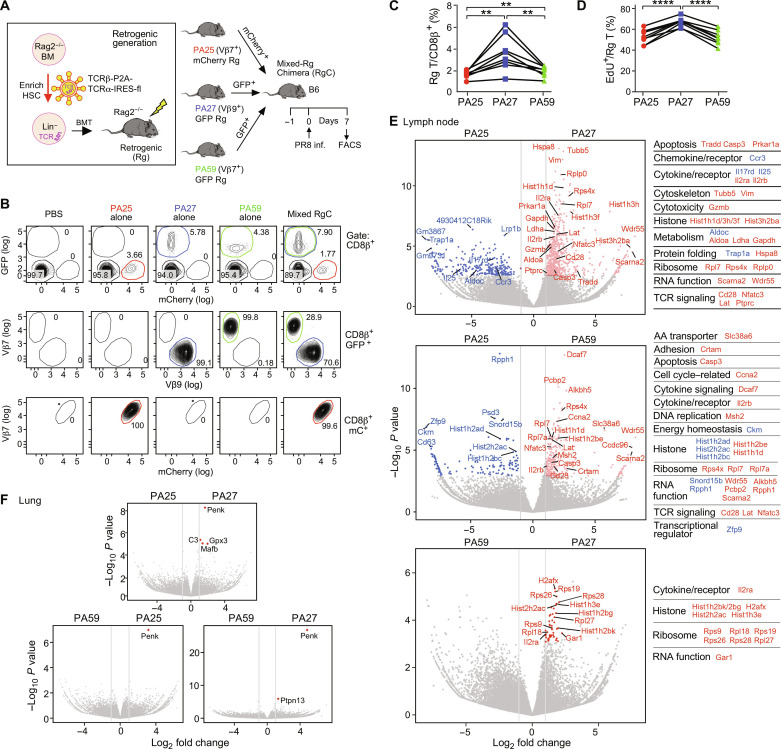
In vivo transcriptomes and expansion of T cells expressing digital TCRs during the acute IAV response. (**A**) Experimental schema to analyze in vivo behaviors of three distinct clonotypic PA_224–233_/D^b^ TCRs in the same mice. Rg mice were generated by transferring *Rag2*^−/−^-derived HSC after transduction of retroviruses containing TCRβ-P2A-TCRα with an FP gene into irradiated *Rag2*^−/−^ mice (left). Subsequently, mixed RgC mice were generated by adoptively transferring an equal number of naïve FP^+^CD8β^+^CD44^−^ T cells from PA25-mCherry, PA27-GFP, and PA59-GFP Rg mice into recipient B6 mice, followed by PR8 infection of the latter 24 hours posttransfer. Rg T cells were analyzed on day 7 post-infection. (**B**) Representative contour plots showing the frequency of Rg T cells in mLN of mixed RgC mice. PA25 T cells were identified as CD8β^+^mCherry^+^Vβ7^+^ (TRBV29^+^) cells (red circle), PA27 T cells as CD8β^+^GFP^+^Vβ9^+^ (TRBV17^+^) (blue circle), and PA59 as CD8β^+^GFP^+^Vβ7^+^ (TRBV29^+^) (green circle) by flow cytometry. (**C**) Quantification of the frequency of Rg T cells in mLN. (**D**) Quantification of the percentage of EdU^+^ Rg T cells. (**E**) Volcano plots of bulk RNA-seq expression data of FACS-isolated clonotypes displaying differentially expressed genes (DEG) as colored dots (red or blue) between PA25 and PA27 Rg T cells (top), PA25 and PA59 (middle), and PA59 and PA27 (bottom) in mLN. Each gene of note is functionally categorized and listed on the right. (**F**) Volcano plots indicating the paucity of DEG between each Rg T cells in lung. For (C) and (D), data are representative of four independent experiments. *P* values were calculated by paired *t* test. **P < 0.01 and ****P < 0.0001. For (E) and (F), DEG (the red and blue dots) are identified as log_2_ fold change > 1 (gray vertical lines) and adjusted *P* value ≤ 0.05 (Wald test with Benjamini-Hochberg correction). BMT, bone marrow transplant; AA, amino acid; HSC, hematopoietic stem cells.

To assess the impact of differential digital TCR performance in vivo, we generated Rg mice by transferring *Rag2*^−/−^ hematopoietic stem cells (HSC) retrovirally transduced with each TCRαβ clonotype and an internal ribosomal entry site (IRES)–linked fluorescence protein (FP) into recipient *Rag2*^−/−^ mice (Fig. 4A). Subsequently, an equal number of naïve Rg-T cells from those Rg mice were adoptively transferred into B6 mice [Rg-chimera mice (RgC mice)] followed by PR8 infection (Fig. 4A and fig. S8A). FACS analysis allowed for detection of the Rg-T cells using a combination of the fluorescent protein and antibodies against Vβ [green fluorescent protein–positive (GFP^+^) V9β^+^ for PA27, GFP^+^ Vβ7^+^ for PA59, and mCherry^+^Vβ7^+^ for PA25) and revealed that the dominant Rg-T cells in mLN are PA27, followed by PA59, and then by PA25 at 7 days postinfection (7 dpi) (Fig. 4, B and C). Those data are consistent with increased in vivo EdU incorporation by PA27 relative to PA25 and PA59 that was not significantly different from one another (Fig. 4D and fig. S8B). Of note, percentages of all three Rg T cells relative to CD8β^+^ T cells were equivalent in lung (fig. S8, C and D).

To exclude the possibility that the binding of mAbs to the TCRs used for cell sorting might have induced TCR signaling and affected gene expression, we used a third FP blue fluorescent protein (BFP), for PA27, thus generating an “untouched” TCR labeling and sorting system. Subsequently, we generated mixed RgC mice and performed bulk RNA sequencing (RNA-seq) at 7 dpi (fig. S9A and data S4). Two of the 12 samples, one from PA25 mLN and one from PA59 lung, were excluded from further analysis due to low RNA quality. Principal components analysis shows that each Rg T cell type in mLN is clustered, whereas those in lung are scattered and undistinguishable (fig. S9B). Compared to PA25 and PA59, PA27 T cells differentially up-regulate genes (635 and 48 genes, respectively), including those involved in TCR signaling, cytotoxicity, cytokine and chemokine receptors, ribosomes, metabolism, and apoptotic genes (Fig. 4E and data S5). Gene set enrichment analysis (GSEA) also revealed that cell cycle pathways are significantly up-regulated in PA27 compared to both PA25 and PA59, and the TCR signaling pathway is additionally up-regulated in PA27 T cells compared to PA25 (fig. S9C). Compared to PA25, PA59 T cells up-regulate 264 genes, including TCR signaling, ribosome, and cell cycle genes (Fig. 4E), although the GSEA was not statistically significant (fig. S9C).

In contrast to the many differentially expressing genes among the three PA-Rg T cells in mLN, there were almost no up-regulated genes in those same PA-Rg-T cells in lung, except for a few most prominently displayed in PA27 T cells such as *Penk*, *Mafb*, *Gpx3*, and *C3* (Fig. 4F and fig. S9D). The overall comparability of the three types of Rg T cells in lung clearly is not due to their unresponsiveness because all significantly up-regulated genes associated with TCR signaling, inflammation, and cytotoxicity compared to those in mLN including *Nur77*, *Zap70*, *Nfat*, *Gzmb*, *Prf1*, and *Il2ra* (fig. S10, A to E). The equivalence of gene expression in the lung is probably a consequence of the collective T cell activation resulting from αβTCR triggering in the context of inflammation with attendant cytokine- and chemokine-mediated signaling spawning modest but equivalent antiviral activity in those PA-Rg T cells (fig. S10F). Of note, ex vivo killing assay shows no PA-Rg T cell–mediated cytotoxicity of LET1 cells (fig. S10G), most likely because of the virtual absence of PA peptides presented on a major fraction of LET1 cells (fig. S1C) compared to, on average, a 5- to 10-fold higher number on the DC2.4 dendritic cell line postinfection (*34*). Thus, it is probable that PA-T cells expand by recognizing PA_224–233_/D^b^ presented on DC in mLN and contribute to virus clearance in lung through production of cytokines and chemokines.

## DISCUSSION

Over 200 million years of jawed vertebrate (*Gnathostomata*) evolution, mammals have developed an αβT cell lineage immune system that uses mechanosensing to detect sparse pMHC ligands. This advancement enhances sensitivity by 1000- to 10,000-fold compared to T cells operating without bioforces (*15*). The Cβ FG loop plays a crucial role by stabilizing the Vβ-Cβ domain interaction, controlling pMHC interaction surface orientation, and contributing to TCR specificity, sensitivity, and bond lifetime (*17*, *25*). Load applied across the TCR-pMHC interface stabilizes interdomain contacts both within the TCRαβ domains and with pMHC, thereby fostering access to transitioning between compact and extended TCRαβ conformations, sustained by external force arising from actomyosin machinery in the T cell and APC (*15*, *16*). As expected, deletion of the Cβ FG loop markedly degrades αβTCR-pMHC recognition function (*17*). In contrast to αβT cells, γδT cells lack the equivalent of the elongated Cβ FG loop, as it is apparently unnecessary for their recognition of abundant nonpeptidic surface ligands. Consequently, γδT cells manifest neither catch bonds nor structural transitions (*40*). These findings fill a gap in the understanding of early components of TCR-mediated T cell activation previously investigated more broadly [reviewed in (*16*)].

Here, we show that with proper force feedback, the TCR-pMHC bond can adopt an unprecedented resonant state revealing lifetimes 10 times greater than peak catch bond lifetimes (Fig. 3). Given the fixed separation between traps, which includes the TCR-pMHC bond, DNA linkage, beads, and optical springs (with physiologically relevant stiffnesses in the 0.2- to 0.3-pN/nm range), a sudden increase in length of the TCR-pMHC bond results in transient relaxation and corresponding reduction of force. This, in turn, shifts the energy landscape to just below the equilibrium force, thereby favoring transition back to the compact state and a reset of the cycle. Unlike protein unfolding where the distance to the transition state in the forward direction, i.e., unfolding, is very short (in the angstrom or single nanometer range) compared to the multiple nanometer-scale refolding distance and thus energetically unlikely to refold with a sustained load (*41*, *42*), the nanometer-level forward and reverse TCR-pMHC transition state distances are more balanced. This greater parity fosters the rapid volleying observed within a narrow “resonant window” of the critical force, a strategy that may apply to other receptor systems.

**Fig. 5.**
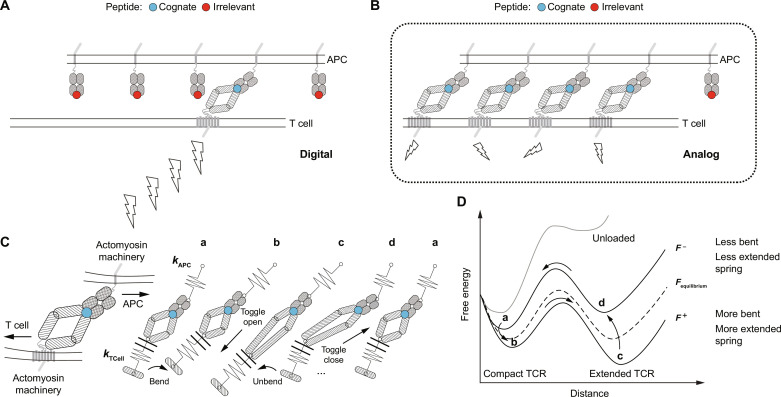
Depiction of the dynamic energy landscape cycle for sustained signaling associated with TCR molecular resonance. (**A**) Depiction of digital TCRs that strongly activate T cells (coordinated bolts) with sparse cognate pMHC on APCs. (**B**) Analog TCRs whose T cells require many copies of the same ligands for activation (distributed bolts) by contrast with digital TCRs. (**C**) The TCR-pMHC interaction results in mechanical connectivity between the T cell and APC at the cell-cell interface with force generated through their respective actomyosin machineries. Depiction of molecular volleying transitions (a to d) near the equilibrium force. Mechanical coupling is illustrated as a “spring” in series that will be viscoelastic in reality. Applied forces are slightly higher in states b and c and slightly lower in states a and d due to extension of the complex. (**D**) Dynamic energy landscape cycle. Dashed line, equilibrium case when the two states have equal free energy at force *F*_equilibrium_. When a slightly higher force (F^+^) is applied, the energy landscape tilts to the right, so that the extended state c is preferred (b → c transition). The resulting extension of the complex relaxes force below the critical level (F^−^) where the energy landscape tilts leftward, and c → d and then d → a transitions can follow, completing a cycle.

A T cell and APC conjugate create a similarly constrained organization (Fig. 5, A and B) that drives bending and unbending of the membrane (*43*) with lateral agitation as the TCR toggles open and closed upon a dynamic energy landscape (Fig. 5, C and D). Each cycle represents a means to initiate T cell activation. Thus, what matters for digital T cell performance is not necessarily the presence of a catch bond per se but rather energetically driven signaling through TCR molecular resonance powered by cell surveillance motions and sustained cycles of conformational changes. Such motions capable of exerting interfacial forces of an appropriate magnitude have been seen in prior work [(*44*) and reviewed in reference (*16*)]. The trap spring constant *k* used here, ~0.25 pN/nm, permits the repeat toggling between the two energy landscape states which is well within the ~1-pN/nm range measured by Hong *et al.* (*45*) for the stiffness of the heterodimer on a cell. The catch bond itself may serve more as a gating mechanism to exclude unproductive interactions such as self-reactivity. Bonds that survive this prescreen may then become energized and support molecular resonance with attendant downstream signaling. Local stiffness and other mechanical elements such as the surrounding accessory and adhesion molecules can tune the resonant cycle. Considering that CD8 binds to the side of the MHCα3 domain, the volleying of the TCR will result in a differential yank at the membrane through pMHC-CD8 linkage(s) with potential to repeatedly and simultaneously drive both inside-out and outside-in signaling (*45*). Bear in mind that the current SMSC data with high-performance digital TCRs have less reliance on CD8, at least for the initial engagement, consistent with prior studies (*17*). How adhesion pairs involving CD2 and LFA-1 and their ligands affect volleying in the immunological synapse that forms subsequently (see below) also deserves further study.

Figure 5 (C and D) depicts the dynamic energy landscape involving the TCR-pMHC interaction per se. Force across the bond pulls the system from state “a” to state “b,” bending the local membrane and shifting the equilibrium to where transitioning to state “c” is favored. Transitioning from b to c, the TCR toggles open (shown as an extension of the constant domain) which extends the bond leading to agitation of the membrane. The system immediately adjusts to the new contour length reducing tension on the springs and unbending to state “d.” The force is now slightly lower than that at equilibrium, reducing tilt of the energy landscape such that the TCR toggles back to state a which retracts the tether agitating the membrane. Cell motions, in turn, pull the bond to state b to allow a repeat of the cycle. Feedback between leftward motions such as the T cell motion indicated (or retrograde flow, not shown therein) and rightward motion such as from an opposing APC motion (or internal motor activity along the cortical actin, not shown) sustains the cycle through multiple repeats, setting up a form of resonance. In analogous SM_db_ assays, changes in the contour length due to TCR-pMHC bond extension reduce force across the tether in the optical trap driving the system between a compact higher force state and extended lower force state. Note that without loading of the TCR-pMHC bond (unloaded curve), the energy barrier is too great to overcome. Under load, in contrast, the forward and reverse distances to transition states are relatively balanced, facilitating the dynamic energy landscape cycle. Ergo, the landscape and attendant TCR function is dependent on bioforces. The function of volleying in the cell context shall be exciting to explore in future studies as a consequence of the current set of observations.

That digital versus analog performance can be determined by a single amino acid in CDR3 at the αβTCR-pMHC interaction surface (Fig. 1) highlights the impact of the dynamic interactions involved in physiologic cognate recognition processes. Consistent with this notion, single amino acid changes in a peptide (i.e., agonist versus antagonist or null) result in disparate activation of T cells expressing the same TCR (*46*). Yet, the TCR-pMHC complexes are virtually identical in static x-ray crystallographic snapshots of those interactions (*47*). We recently found that asymmetric interdomain motion and interfacial contacts under load affect the peptide-sensing CDR3 loops, thereby determining mechanical response and peptide discrimination (*28*).

In a digital response, repetitive transitions of single αβTCRs can activate motor proteins to foster kinapse initiation and subsequently mature immunological synapse formation. Both OT and super-resolution microscopy experiments revealed that pMHC-ligated αβTCRs and nearby unligated αβTCRs are recruited to initiate TCR clustering (*15*, *48*). Analog TCRs, on the other hand, may benefit from a high density of pMHC ligands to allow for integration of signal or coalescence of smaller TCR clusters as an alternative means to synapse initiation. Regarding TCRs targeting NP_366–374_/D^b^, both digital and analog expressing T cells can be engaged productively given the high copy number of that ligand displayed on IAV-infected epithelium. Integration of signals from multiple TCRs can afford sensing across a ligand gradient and is presumably operative in T cells bearing either analog or digital TCRs. By contrast, digital TCRs can recognize sparse ligands like PA_224–233_/D^b^ where analog TCRs cannot interpret such rare input to drive T cell signaling.

PA TCRs showed distinguishable biophysical performances and activation responses in different functional assays (Figs. 2 and 3). The PA T cell transcriptomes were distinct, consistent with the ability of calcium flux and prolonged ERK activation to affect T cell expansion and gene activation (*49*, *50*). That PA27 has high level of transcripts encoding the antioxidant Gpx3 and proenkephalin (an attenuator of substance P that promotes asthma via the PI3K/Akt/Nfκb pathway in bronchial epithelium) (*51*) speaks to a potential protective effect mediated by this T cell in lung. The distinct bond lifetime occurring at higher force for PA59 relative to PA27 and PA25 is noteworthy since local tissue stiffness varies 100-fold in normal versus inflammatory conditions and even more profoundly among different tissue types (*52*–*54*). The increased breadth of the PA59 catch bond curve may capitalize on accessing higher force interactions expected in stiffer tissues where a bond captured at ~20 pN can persist long enough to subsequently relax to a resonant state. Perhaps PA59 functions best in stiff locales such as intraepithelial sites where certain resident memory T cells reside. That mLN PA59 T cells express higher *Itgae* (CD103) and *Itgb7* transcripts than PA27 (data S4) may enhance αEβ7 integrin expression to facilitate intraepithelial site localization through E-cadherin counter-receptors (*55*). Although there is not a universal bioforce load for all αβTCRs (Fig. 2B), the PA25 performance is least ideal among the three PA TCRs examined. Nevertheless, in vivo studies have revealed that PA25 function in the lung is broadly comparable to that of the other TCRs, emphasizing how acute inflammation-related cuing fosters productive responses to benefit the host. In future studies, in vivo kinetic analysis of digital versus analog Rg TCRs will be important in determining temporal acquisition of CTL phenotypes in the lung for both PA and NP specificities.

Our discovery of digital-versus-analog performances coupled with distinct bioforce profiles has broad implications. We have identified various quantitative TCR performance metrics including the parameterization of catch bond curves, conformational transitions, critical force and lifetime of volleying, tether formation, activation threshold, and signaling profiles. In addition, our findings suggest opportunities for nuanced adoptive T cell immunotherapies and/or cancer vaccine elicitation of relevant TCR specificities with digital TCR performance requirements mandated by sparse neoantigen arrays on cancer cells. The stiffness of desmoplastic solid tumors such as pancreatic cancers likely necessitates using TCRs with greater force-bond lifetime maxima than would compliant hematopoietic tumors.

Connors and colleagues (*56*) recently uncovered an important clue about differential TCR performance linked to CTL vaccine failure in the setting of infectious disease. They demonstrated that an adenovirus 5 (Ad5)–based HIV-1 vaccination strategy in high-risk normal volunteers [Self-Testing Education and Promotion (STEP) trial] was unable to induce CD8 T cells with performance capable of facile CTL-mediated degranulation and polarization, unlike CTL of identical pMHC specificities derived from long-term nonprogressors (LTNP) naturally infected with HIV-1. The LTNP group controls viral load via their own immune response. In contrast, vaccination of STEP trial volunteers neither offered protection against subsequent HIV-1 exposure nor prevented HIV-1/AIDS progression. cDNA cloning and transfer of these two groups’ αβTCRs into normal T cells recapitulate the respective behaviors, demonstrating that the differential cognate antigen recognition function is determined by the TCRs per se. We suggest that Ad5 vaccine likely induces analog, not digital TCR responses, perhaps consequent to a high copy number of the specific pMHC on APCs directed by the Ad5 vector. This contrasts to natural HIV-1 infection that generates a low copy pMHC array (*56*–*58*). On the other hand, sparse ligand array almost certainly focuses the LTNP T cells into a digital performance mode, akin to the low copy number PA_224–233_/D^b^ pMHC mouse IAV T cell response observed herein. If our hypothesis is correct, then the results imply that the density of pMHCs used for stimulating an effective response via vaccination should be limited when that on a pathogen-modified cell or tumor cell is sparse.

Biophysical parameterization and functional activation assays in tandem suggest that it is possible to uncover biomarkers of significance. Of note, surface CD3 loss and CD69 up-regulation observed here were shown to be associated with TCR quality in a recent independent study (*59*). Our transcriptomic analysis advocates that cytokine receptors and elements involving the cytotoxicity machinery, ribosomes, RNA regulation, and TCR signaling (Fig. 4E) might all be facile biomarker candidates. Defining the structural basis of TCR performance is worthy of future investigation and may lead to potent synthetic TCR designs. Using molecular dynamics in conjunction with machine learning could achieve the aspirational goal of predicting each TCR’s performance within a T cell repertoire specific for a given pMHC under various physical loads based on primary sequence information.

## MATERIALS AND METHODS

### Mice and IAV infection

C57BL/6N (B6), B6.129S6-Rag2^tm1Fwa^N12 (*Rag2*^−/−^), and B6.SJL-*Ptprc^a^*/BoyAiTac (CD45.1) mice were purchased from Taconic Biosciences Inc., housed and bred under specific pathogen–free condition at the Dana-Farber Cancer Institute Animal Facility, accredited by the Association for Assessment and Accreditation of Laboratory Animal Care. Euthanasia was performed by CO_2_ inhalation followed by cervical dislocation. Sex-matched mice were used for each experiment. No gender preference was expressed for this study, and the gender in each experiment was not deliberately selected. Mice at 6 to 10 weeks of age were infected intranasally with 3 × 10^4^ egg infectious dose (EID)_50_ of influenza A/PR/8/34 virus (PR8, H1N1, Charles River Laboratories) as a primary infection under anesthesia with intraperitoneally injection of ketamine/xylazine [ketamine (120 mg/kg) and xylazine (10 mg/kg)]. PR8-infected mice were rechallenged intranasally with 5 × 10^7^ EID_50_ of serologically distinct X:31, A/Aichi/68 (X31, H3N2, Charles River Laboratories). For detecting cell proliferation in vivo, mice were intraperitoneally injected with 0.5 mg of EdU (Invitrogen) 3 hours before sacrificed. All mouse maintenance, breeding, and experimental procedures were approved under Dana-Farber Cancer Institute Institutional Animal Care and Use Committee protocol #04-113.

### Cell lines and cell culture

BW5147.3 cells and CD3δγεζ pMIY vector were gifts from the Vignali lab (St. Jude Children’s Research Hospital, Memphis, TN), and mCD8αβ^+^ BW5147.3 cells were generated as previously described (*20*). The cells were maintained in D10 [Dulbecco’s modified Eagle’s medium (DMEM) medium (Gibco), 10% fetal bovine serum (FBS) (Sigma-Aldrich), 100 IU penicillin and streptomycin (100 μg/ml; Corning), 2 mM l-glutamine (Corning), and 55 μM 2-mercaptoethanol (Gibco)], with hygromycin B (400 μg/ml; Gibco) and Geneticin (400 μg/ml; Gibco). R8 cells were a gift from the Glimcher lab (Harvard School of Public Health, Boston, MA) and maintained in R10 [RPMI 1640 (Gibco), 10% FBS, 100 IU penicillin and streptomycin (100 μg/ml), and 55 μM 2-mercaptoethanol]. The retroviral packaging cell line, Plat-E, was purchased from Cell Biolabs and were grown in D10 with puromycin (1 μg/ml; Sigma-Aldrich) and blasticidin (10 μg/ml; Gibco). LET1 cells were obtained from BEI Resources and maintained in D10. All cells were grown in a 5% CO_2_ incubator at 37°C.

### Cell isolation

Resident CD8^+^ T cells from lung were isolated as previously described (*60*). Briefly, mice were intravenously injected with 0.8 μg of phycoerythrin (PE)– Cyanine 7 (Cy7)–conjugated anti-CD8α mAb in 200 μl of phosphate-buffered saline (PBS) 5 min before euthanasia to distinguish CD8^+^ T cells residing in lung tissue from those in lung vasculature (*61*). Subsequently, lung blood vessels were gently perfused with 60 ml of PBS through the right ventricle to wash out the residual injected antibody, and then lung tissues were harvested. After mincing lungs with scissors, the chopped tissues were digested with collagenase D (2 mg/ml) and deoxyribonuclease I (80 U/ml) in Hanks’ balanced salt solution (HBSS) at 37°C for 1 hour with manual rotation every 10 min. Digested tissues were dissociated by gentleMACS Dissociator (Miltenyi Biotec), and the cells were filtered through a 70-μm cell strainer. Red blood cells were eliminated from the cell suspension by treating with hemolysis buffer [140 mM NH_4_Cl and 17 mM tris-HCl (pH 7.2)]. Cell suspensions were resuspended with FACS buffer (2% FBS/0.05% NaN_3_/PBS) for the following experiments. mLN was harvested, mashed with a 3-ml syringe on a 24-well plate with RPMI 1640, filtered with 80-μm mesh, washed with RPMI 1640, and resuspended with FACS buffer.

### Flow cytometry and cell sorting

Cell suspensions from tissues were first treated with anti-mouse CD16/CD32 mAbs in FACS buffer to block FcR binding for 10 min at 4°C and then stained with antibodies indicated in each figure legend in FACS buffer for 20 min at 4°C. All antibodies and the concentration used are listed in data S6. For single-cell RT-PCR, the cell suspension from pooled lungs from two mice was stained with APC-conjugated PA_224–233_/H-2D^b^ tetramer (MBL International Corporation) and PE-conjugated NP_366–374_/H-2D^b^ tetramer (MBL International Corporation) for 30 min at room temperature followed by staining with CD8β mAb to discriminate tissue-resident CD8 T cells defined as CD8α^−^CD8β^+^ cells which were spared from vascular CD8α^+^CD8β^+^ cells. PA_224–233_- and NP_366–374_-tetramer^+^ cells were concurrently sorted after gating on 7-aminoactinomycin D^−^CD8α^−^CD8β^+^ cells. For RNA-seq, mCherry^+^ (PA25), BFP^+^ (PA27), and GFP^+^ (PA59) cells were simultaneously sorted after gating on Zombie-NIR^−^ CD8α^−^CD8β^+^ cells.

EdU staining was performed using Click-iT Plus EdU Alexa Fluor 647 or Pacific Blue Flow Cytometry Assay Kit (Invitrogen) following the manufacturer’s instructions. For intracellular Ki67 staining, cells were fixed with 4% paraformaldehyde (PFA)/PBS, treated with a permeabilization buffer [0.1% saponin (Sigma-Aldrich) in FACS buffer], and incubated with APC-anti mouse-Ki67 mAb at 4°C for 30 min. For detecting NP protein in LET1 cells, the cells were infected with PR8 in Opti-MEM (Gibco) for 1 hour, and then the inoculum was washed out with washing buffer (5 mM Cacl_2_/5 mM MgCl_2_/20 mM Hepes/HBSS) followed by culture in R10 for 24 hours. Intracellular NP staining was performed using the BD Cytofix/Cytoperm Fixation/Permeabilization Kit (BD Biosciences) and a fluorescein isothiocyanate–conjugated influenza A NP mAb (Invitrogen). Cells were analyzed on a BD LSRFortessa Cell Analyzer (BD Biosciences) or sorted by using FACS Aria II (BD Biosciences). Data were analyzed with FlowJo software (FlowJo LLC).

### Single-cell RT-PCR

Single PA_224–233_- or NP_366–374_-specific cells were sorted to each well of a 96-well plate that contained 3 μl of RT mix1 (data S7). Amplification of cDNA of TCRα or TCRβ was performed using single-cell RT-PCR method as previously described (*62*, *63*). All the PCR primers and the component of all the reaction mixtures are listed in data S6. To perform the RT reaction, 2 μl of the RT mix2 was added to each well containing a single T cell. After incubation for 60 min at 42°C, 15 μl of the first PCR mix was added to each well to perform the first PCR reaction. The program for the first PCR reaction was as follows: 1 min at 98°C followed by 30 cycles of 10 s at 98°C, 5 s at 53°C, and 40 s at 72°C. The resultant first PCR products were diluted 10-fold with nuclease-free water (Invitrogen) and used for a second cycle of PCR. In the second cycle, TCRα and TCRβ were amplified separately. To amplify the cDNA of TCRα or TCRβ, 2 μl of the diluted first PCR products was added to each well of a new 96-well PCR plate containing 18 μl of the second PCRα mix or the second PCRβ mix, respectively. The program for the second PCR reaction was as follows: 1 min at 98°C followed by 35 cycles of 10 s at 98°C, 5 s at 58°C, and 30 s at 72°C. The second PCR products were then analyzed with the Ca_RV3 primer for TCRα or Cb_RV3 primer for TCRβ by direct sequencing. The TCR repertoire was analyzed with reference to the ImMunoGeneTics database (www.imgt.org).

### Clonotype definition

Assembled TCR genetic elements yielded nucleotide sequences encoding a subunit clonotype for each cell. TCRα and TCRβ clonotypes were counted separately, and those clonotypes with productive CDR3s were selected for analysis. Each paired TCRαβ clonotype unique to a given single cell was of particular functional interest. The hierarchy of subunit clonotypes is shown in the pie charts in Figs. 1A and 2A. TRV and TRJ repertoires and CDR3 sequence for all TCR clonotypes are listed in data S1.

### Retrovirus production and transduction

cDNA encoding TCRβ-P2A-TCRα was inserted into a retroviral vector pMSCV-IRES-GFP II (pMIG II, Addgene, #52107), pMSCV-IRES-mCherry FP (Addgene, #52114), or pMSCV-IRES-Blue FP (Addgene, #52115). For the real-time killing assay, nonfluorescence TCRαβ-expressing vectors were made by cutting IRES–enhanced GFP site out from pMIG II with restriction enzymes and by inserting TCR cDNA. The vector was transfected into Plat-E cells with Fugene HD (Promega). Retrovirus in the cultured supernatant was collected 72 hours later and frozen at −80°C until use. Thawed retrovirus was transduced with RetroNectin (Takara Bio) using RetroNectin-bound virus infection methods according to the manufacturer’s instruction. Retrovirus was transduced into mCD8αβ^+^ BW5147.3 cells to generate TCRαβ-expressing cell lines or into mouse bone marrow (BM) cells to generate Rg mice. To establish TCRαβ-expressing BW cell lines, the transduced cells were sorted by FACS Aria II (BD Biosciences) to match the TCR expression based on the cell surface expression of CD3ε. Before all assays were performed, CD3 or TCRβ surface level was confirmed to be matched in a group.

### Generation of Rg mice

Rg mice were generated as previously described (*64*). Briefly, BM cells were harvested from *Rag2*^−/−^ mice, and HSC were enriched by the EasySep Mouse Hematopoietic Progenitor Cell Isolation Kit (STEMCELL Technologies) followed by expansion in Stem cell medium [StemPro-34 serum-free medium (Gibco), 5% FBS, 100 IU penicillin and streptomycin (100 mg/ml), and 2 mM l-glutamine, with mouse IL-3 (50 ng/ml; STEMCELL Technologies), human IL-6 (50 ng/ml; STEMCELL Technologies), and mouse stem cell factor (50 ng/ml; STEMCELL Technologies)] for 3 days. Subsequently, HSC were transduced with retrovirus encoding TCRαβ, cultured in Stem cell medium for 3 days, and then transferred into *Rag2*^−/−^ mice irradiated with Gamma Cell 40 Cs^137^ Irradiator (Thratonics) 1 day before. Rg mouse blood was analyzed for CD8 development by flow cytometry 6 weeks after transplantation, and the mice expressing more than 10% of CD8 T cells in CD45^+^ cells were used for generation of RgC mice.

### Generation of RgC mice

For single RgC mice, peripheral LNs and spleen were harvested from Rg mice, and FP^+^CD8β^+^CD44^−^ naïve T cells were sorted. Subsequently, 2 to 10 × 10^4^ cells were intravenously transferred into recipient B6 mice. For mixed RgC mice, an equal number of Rg T cells were mixed before transfer. The ratio of the mixed Rg T cells was confirmed by the combination of FP and Vβ expression by flow cytometry. RgC mice were intranasally infected with PR8 1 day after adoptive transfer. For real-time killing assay, CD8β^+^CD44^−^ naïve T cells from non-FP Rg mice were sorted and adoptively transferred into recipient CD45.1 mice.

### TCR protein expression

PA_224_-specific TCRαβ or NP_366_-specific NP41αβ LZ proteins were produced and used for SM assay as previously described (*17*, *65*), Briefly, separate chains were expressed in Expi293F (Thermo Fisher Scientific) cells according to the manufacturer’s protocol and purified from supernatants as a LZ paired heterodimer using an anti-LZ mAb (clone 2H11). The TCRαβ constructs consist of V and C ectodomains connected to the 30–amino acid LZ motif via a 15-residue flexible linker sequence. The heterodimer was covalently linked via the native disulfides located at the C-terminal end of each ectodomain.

### SMSC assay

SMSC assay was performed to measure the specific bond lifetime of TCR-pMHC interaction using an SM DNA tether, which was functionalized with a half anti-biotin antibody to capture biotinylated mutated pMHC at one end and with a digoxigenin tag for tether adhesion on anti-digoxygenin–coated polystyrene beads (1.0 μm in diameter, Spherotech Inc.) at the other end. The bead slurry was washed with PBST buffer [1× PBS + 0.02% (v/v) Tween 20] twice and then resuspended with a dilution factor of 200× in colorless DMEM medium supplemented with bovine serum albumin (5 mg/ml; BSA) for bond lifetime measurements. Cells used in SMSC assay were rinsed once with colorless 1 ml of DMEM medium and resuspended to a final concentration of 2 × 10^6^ cells/ml. Next, 20 μl of this cell suspension was transferred into the flow chamber, where the cells were allowed to attach to a polylysine-coated coverslip. The chamber was then incubated at 37°C with 5% CO_2_ for 30 min. Afterward, the coverslip surface was passivated using colorless DMEM medium supplemented with BSA (5 mg/ml), followed by a 10-min incubation at 37°C with 5% CO_2_. Subsequently, approximately 20 μl of pMHC-tethered bead slurry was pipetted on one side of the flow chamber and sucked out the other side by capillary action using a Kimwipe. The pMHC-tethered bead was trapped by the trapping laser (1064 nm) and brought close to a nearby cell, resulting in the formation of a stable tether between the bead and T cell. Pulling force was generated by stepping the piezo stage with a defined distance in the direction opposite to the approaching direction. Specific procedures used for preparing the beads and measuring bond lifetime are described in a previous work (*17*).

### SCAR assay

SCAR assay was performed to measure early T cell activation via staining the cells with Quest Rhod-4, AM (AAT Bioquest Inc.) to visualize the intracellular Ca^2+^ flux. Subsequently, WT pMHC-coated beads with varying interfacial copy numbers were used to assess the triggering capability for different T cell lines. Beads were trapped and associated with the cell for a minimum of 5 s. Attachment was verified by briefly turning off the detection and trapping lasers. Compared to the normal forces in SMSC assay, tangential forces parallel to the cell-bead interface were applied with defined magnitude. Detailed protocols for the preparation and quantification of interfacial number of molecules of pMHC-coated beads, Quest Rhod-4 staining, and intracellular Ca^2+^ activation with optically trapped beads can be found in a previously published work (*15*). For Figs. 1D and 2D and figs. S2 (F and G) and S5 (B and C), adaptations made to the protocol include the following: (i) Streptavidin beads with a diameter of 1.36 μm (Spherotech Inc.) were used; poly-l-lysine–coated coverslips were used to facilitate cell binding to the coverslip surface; flow channels were formed with a single layer of double-sided tape; and fluorescence images were taken every 5 s for 10 to 15 min.

SCAR experiments performed for Figs. 1D and 2D and figs. S2 (F and G) and S5 (B and C) were acquired using a microscope adapted for combined trapping and SM fluorescence with low levels of fluorescence excitation. In these experiments, we used a trapping laser power of ~350 mW and total fluorescence excitation laser power of 5 μW in Epi mode with an excitation zone spread over an area of ~2827 μm^2^ (~1.77 × 10^−3^ μW/μm^2^). For Fig. 2D, individual curves from all triggering cells from each respective cell line were pooled (combining interfacial pMHC concentrations of 20 and 2 with force for NP63, NP34, NP41, PA25, PA27, and PA59 and 2 with force for N15) and averaged at time 0. All curves fit to *y* = A*(1 − *e*^−*x*/*t*^), where *y* is the fluorescence intensity, *A* is the amplitude, *t* is the rise time constant (s), and *x* is the time (s) (dashed line). Detailed information is in data S3. N15 recognizes VSV8/K^b^ (*14*–*17*), unlike the NP and PA TCRs which bind NP_366–374_/D^b^ and PA_224–233_/D^b^, respectively. One-way analysis of variance (ANOVA) performed on the amplitudes of digital cells versus analog tests shows a one-star significance (*P* value = 0.03).

### SM assay

Purified single heterodimers with LZ as described above coated one bead via 2H11, which was covalently linked to 1.23-μm polystyrene beads (Spherotech Inc.) via 1-ethyl-3-(3-dimethylaminopropyl)carbodiimide chemistry. A 3500-bp DNA tether with digoxigenin on one end and streptavidin on the other was used to link biotinylated mutant PA_224–233_/H-2D^b^ or NP_366–374_/H-2D^b^ monomer to a second 1.23-μm polystyrene bead coated with anti-digoxigenin. Streptavidin was used to connect limiting amounts of pMHC to the DNA tether. Beads were loaded into separate channels of the microfluidic system on the LUMICKS m-Trap. Each bead was trapped in respective channels and moved to empty PBS channels where they were calibrated, brought together to form tethers, and were rapidly pulled apart to load a known force. Beads were used for three to seven lifetime measurements before discarding and trapping a new bead pair. Trap stiffnesses ranged from 0.20 to 0.30 pN/nm. Interactions were measured until bond rupture. In the case of volleying, the trap separation could be slightly adjusted by a few nanometers to cause fraction of a pN changes in force to keep the system within the volleying force range. Changes in positional distribution could be directly observed as a result of said minute adjustments (Fig. 3, H and I). In controls lacking TCR and pMHC with DNA of the same length and biotin and digoxigenin functionalization, no conformational transitions were observed. Hopping frequency versus force were evaluated for 10-s segments except for NP41 which were shorter due to the lack of sustained volleying (Fig. 3F). Transitions between the two major dwell states are considered while excluding transitions less than 5 nm (Fig. 3G).

### Tetramer binding assay

A total of 5 × 10^5^ PA-or NP-TCR transduced BW cells were plated on 96-well plates, and titrated WT or CD8BS-mutant PA_224–233_/H-2D^b^ or WT or mutant NP_366–374_/H-2D^b^ tetramer in FACS buffer was added, respectively. The cells were incubated at room temperature for 30 min and washed with FACS buffer twice, and the fluorescence intensity was analyzed by flow cytometry. The tetramers used in each experiment are listed in data S6.

### Tetramer dissociation assay

A total of 5 × 10^5^ PA-BW cells were treated with mutant PA_224–233_/H-2D^b^ tetramer at room temperature for 30 min. WT NP_366–374_/H-2D^b^ tetramer was used for NP-BW cells due to an inability of the mutant tetramer binding for NP41. The cells were washed and plated on 96-well plates. Subsequently, 3 μg of Fab fragment of anti-mouse H-2D^b^/H-2L^d^ antibody was added to the cells for the indicated times at room temperature, and then the cells were immediately fixed in 4% PFA/PBS. The cells were washed, and the fluorescence intensity of the tetramer was analyzed by flow cytometry. The cells without Fab addition were used as a control of time 0. The tetramers used in each experiment are listed in data S6. Fab fragment of anti-mouse H-2D^b^/H-2L^d^ mAb (BioLegend) was made by the Pierce Fab Preparation Kit (Pierce) according to the manufacturer’s instruction.

### Tetramer activation assay

For pERK assay, the cells were resuspended at a concentration of 1 × 10^6^ cells/ml in a final volume of 0.5 ml. For each sample, either the WT PA_224–233_/H-2D^b^ tetramer (0.75 μg/ml) or the WT NP_366–374_/H-2D^b^ tetramer (2.75 μg/ml) was added to the PA- or NP-BW cell samples, respectively. Control samples were also prepared for each cell line without the addition of tetramer at time zero. All cell samples were then incubated on ice for 20 min and then washed with DMEM to remove excess tetramer. The cells were resuspended at a final concentration of 2 × 10^6^ cells per 0.1 ml and incubated at 37°C for the indicated 0- to 10-min time points. Lysis buffer [final concentration after dilution: 1% Triton X-100, 0.05% SDS, 50 mM tris (pH 7.4), 150 mM NaCl, 2 mM NaVO_3_, 1 mM *N*-ethylmaleimide, and Roche cOmplete protease cocktail] was added to the cells, and the samples were immediately placed on dry ice to await further processing. Cell samples were thawed for 10 min on ice and centrifuged at 13 K rpm, 4°C for 15 min, and clarified lysate was transferred to a clean tube. Aliquots were run on 4 to 12% bis-tris NuPAGE gels, transferred to polyvinylidene difluoride membrane for detection with ERK (W15133B clone) and phosphorylated ERK (4B11B69 clone) antibodies, and imaged using the Bio-Rad ChemiDoc imaging system. Band density was measured using the Image Lab software, and the level of phosphorylated ERK was normalized to ERK for each sample. This was then further normalized to the median within each experimental set to mitigate artifacts of antibody staining. Technical replicates were generated for each biological replicate by preparing three sets of cells and conducting at least three biological replicates for each activation assay.

For analysis of CD3ε loss and CD69 up-regulation after tetramer activation, titrated WT PA_224–233_/H-2D^b^ tetramer or WT NP_366–374_/H-2D^b^ tetramer was added to 2 × 10^5^ PA- or NP-BW cells, respectively. The cells were cultured in D10 at 37°C overnight, washed with FACS buffer twice, stained with anti-mouse CD3ε mAb and anti-mouse CD69 mAb, and analyzed by flow cytometry. The gMFI of CD3ε and CD69 without the tetramer was normalized to 100%.

### Cell-based functional avidity assay

A total of 1 × 10^5^ PA- or NP-BW cells were cultured in D10 with titrated PA_224–233_ or NP_366–374_ peptide (from 1 × 10^−5^ to 1 × 10^4^ ng/ml), respectively, at 37°C for 16 to 18 hours overnight. A total of 1 × 10^5^ R8 cells used as APC were treated with mitomycin C (Sigma-Aldrich) and washed with PBS three times before use. IL-2 concentration in the culture supernatant was measured by enzyme-linked immunosorbent assay assay according to the manufacturer’s instruction. EC_50_ of peptide response to each BW cells was calculated by Prism 7 (GraphPad Software).

### Real-time killing assay

To visualize LET1 cells, mCherry retrovirus derived from pMSCV-IRES-mCherry FP vector (Addgene) was transduced. A total of 2 × 10^4^ mCherry^+^ LET1 cells were seeded 18 hours before PR8 infection on 96 well plate (Corning). The cells were washed with washing buffer (5 mM Cacl_2_/5 mM MgCl_2_/20 mM Hepes/HBSS) and infected with titrated PR8 (from 4 × 10^5^ EID_50_ to 4 × 10^8^ EID_50_) in Opti-MEM (Gibco) at 37°C for 1 hour. Rg T cells from mLN or lung were obtained from CD45.1 RgC mice infected with PR8 7 days before and sorted as CD45.2^+^ CD8β^+^ CD44^+^ Zombie Aqua^−^ intravascular stained CD8α^−^. A total of 2 × 10^4^ Rg T cells were labeled with 10 μM Cell Proliferation Dye eFluor 450 (eBioscience) and plated on the infected mCherry^+^ LET1 cells. CellEvent Caspase-3/7 Green ReadyProbes Reagent (Invitrogen) was added to the wells to measure apoptosis. Plates were housed in a BioSpa 8 Automated Incubator (Agilent) and were scanned at regular intervals using a Cytation 5 Cell Imaging Multimode Reader (Agilent). Image processing and data analysis were conducted using the Gen5 3.11 software (Agilent). Whereas the Cell Proliferation Dye eFluor 450 remains bound to apoptotic T cells, mCherry was quickly lost from apoptotic LET1 cells due to intracellular expression of the soluble protein. Therefore, LET1-specific killing was quantified by subtracting T cell–specific apoptotic signal (green and blue overlapping area) from total caspase-3/7 activity levels (total green area). Efficiency of LET1 killing was then calculated via normalization to confluency of viable LET1 cells at each respective timepoint (total red area).

### Measurement of virus titer

Viral copy number was determined by the method previously described (*66*). Briefly, lung tissues were collected, preserved in RNAlater (Sigma-Aldrich), and frozen at −80°C until used. Once thawed, the tissues were homogenized by a tissue disruptor (QIAGEN), and RNA was extracted with a PureLink RNA Mini kit (Invitrogen). Reverse transcription was conducted with the High-Capacity cDNA Reverse Transcription Kit (Applied Biosystems), and quantitative PCR (qPCR) was performed with PowerUP SYBR Green Master Mix for qPCR (Applied Biosystems) and primers specific for NP (forward: 5′-GAT TGG TGG AAT TGG ACG; reverse: 5′-AGA GCA CCA TTC TCT CTA TT-3′) using the 7900HT Fast Real Time PCR System (Applied Biosystems). The standard calibration curve for qPCR was obtained by stepwise dilution of the cloned NP gene fragment with a known copy number.

### RNA sequencing

Following isolation of PA25, PA27, and PA59 Rg T cells from both lung and mLN from three mixed RgC mice for each TCR, each preparation was processed individually (i.e., three biological replicates) for isolation of total RNA using the RNAqueous-4PCR total RNA isolation kit (Thermo Fisher Scientific) that minimizes genomic DNA contamination. Cell input for the LN preparations was 11,643 ± 2486 (mean ± SEM, *n* = 9) and, for the lung preparations, was 45,327 ± 6225 (mean ± SEM, *n* = 9). Quality control was assessed using an Agilent 4200 TapeStation. Libraries suitable for RNA-seq analysis were prepared using the SMART-Seq v4 Ultra low Input RNA kit (Takara), followed by addition of Illumina adapters and 150-bp paired end sequencing on the Illumina NovaSeq 6000 platform (MedGenome Inc.).

### RNA-seq data analysis

Output per library for the LN Rg T cells averaged ~60 M reads and, for the lung resident Rg T cells, averaged ~80 M reads. The paired output fasta files for each sample were checked for quality using FastQC (v0.11.8) and adapters trimmed using FastqMcf (v1.05) and Cutadapt (v4.4). Sequenced RNA-seq reads were aligned to the mouse genome (mm10) using STAR (2.4.2a) (*67*). The RNA-seq pipeline Viper (*68*) was used to generate gene-level read counts and gene-level TPM (transcript per million) values and to perform principal components analysis for the analyzed samples. Differentially expressed genes were identified with the R package DESeq2 (1.38.3) (*69*) using a fold change threshold of 2 and an adjusted *P* value threshold of 0.05. GSEA was performed using the R package clusterProfiler (4.6.2) (*70*). The sources of gene sets used for the analysis are provided in data S8.

### Statistics

Statistical analyses were performed with GraphPad Prism software (v9) except pERK assay and RNA-seq data analysis. Statistical tests used, one-way ANOVA, linear regression, Kolmogorov-Smirnov test, paired *t* test, Wald test, Kruskal-Wallis, or unpaired *t* test, were indicated in figure legends. For three independent experiments of pERK assay, the statistics were performed by regression using trend line analysis models accounting for interexperimental variability [R Statistical Software Package (v4.1.2)].

## References

[1] R. Medzhitov, C. A. Janeway Jr., Innate immunity: Impact on the adaptive immune response. Curr. Opin. Immunol. 9, 4–9 (1997). 9039775 10.1016/s0952-7915(97)80152-5

[2] S. M. Kaech, E. J. Wherry, R. Ahmed, Effector and memory T-cell differentiation: Implications for vaccine development. Nat. Rev. Immunol. 2, 251–262 (2002). 12001996 10.1038/nri778

[3] R. W. Dutton, L. M. Bradley, S. L. Swain, T cell memory. Annu. Rev. Immunol. 16, 201–223 (1998). 9597129 10.1146/annurev.immunol.16.1.201

[4] J. Sprent, C. D. Surh, T cell memory. Annu. Rev. Immunol. 20, 551–579 (2002). 11861612 10.1146/annurev.immunol.20.100101.151926

[5] M. M. Davis, P. J. Bjorkman, T-cell antigen receptor genes and T-cell recognition. Nature 334, 395–402 (1988). 3043226 10.1038/334395a0

[6] C. Brack, M. Hirama, R. Lenhard-Schuller, S. Tonegawa, A complete immunoglobulin gene is created by somatic recombination. Cell 15, 1–14 (1978). 100225 10.1016/0092-8674(78)90078-8

[7] C. Cunningham-Rundles, P. P. Ponda, Molecular defects in T- and B-cell primary immunodeficiency diseases. Nat. Rev. Immunol. 5, 880–892 (2005). 16261175 10.1038/nri1713

[8] S. M. Alam, P. J. Travers, J. L. Wung, W. Nasholds, S. Redpath, S. C. Jameson, N. R. Gascoigne, T-cell-receptor affinity and thymocyte positive selection. Nature 381, 616–620 (1996). 8637599 10.1038/381616a0

[9] M. A. Faust, V. J. Rase, T. J. Lamb, B. D. Evavold, What’s the catch? The significance of catch bonds in T cell activation. J. Immunol. 211, 333–342 (2023). 37459191 10.4049/jimmunol.2300141 PMC10732538

[10] S. C. Meuer, O. Acuto, R. E. Hussey, J. C. Hodgdon, K. A. Fitzgerald, S. F. Schlossman, E. L. Reinherz, Evidence for the T3-associated 90K heterodimer as the T-cell antigen receptor. Nature 303, 808–810 (1983). 6191218 10.1038/303808a0

[11] M. G. Rudolph, R. L. Stanfield, I. A. Wilson, How TCRs bind MHCs, peptides, and coreceptors. Annu. Rev. Immunol. 24, 419–466 (2006). 16551255 10.1146/annurev.immunol.23.021704.115658

[12] J. H. Wang, E. L. Reinherz, The structural basis of αβ T-lineage immune recognition: TCR docking topologies, mechanotransduction, and co-receptor function. Immunol. Rev. 250, 102–119 (2012). 23046125 10.1111/j.1600-065X.2012.01161.x PMC3694212

[13] A. Peri, N. Salomon, Y. Wolf, S. Kreiter, M. Diken, Y. Samuels, The landscape of T cell antigens for cancer immunotherapy. Cancer 4, 937–954 (2023). 10.1038/s43018-023-00588-x 37415076

[14] E. L. Reinherz, W. Hwang, M. J. Lang, Harnessing αβ T cell receptor mechanobiology to achieve the promise of immuno-oncology. Proc. Natl. Acad. Sci. U.S.A. 120, e2215694120 (2023). 37339184 10.1073/pnas.2215694120 PMC10318960

[15] Y. Feng, K. N. Brazin, E. Kobayashi, R. J. Mallis, E. L. Reinherz, M. J. Lang, Mechanosensing drives acuity of αβ T-cell recognition. Proc. Natl. Acad. Sci. U.S.A. 114, E8204–E8213 (2017). 28811364 10.1073/pnas.1703559114 PMC5625899

[16] Y. Feng, E. L. Reinherz, M. J. Lang, αβ T cell receptor mechanosensing forces out serial engagement. Trends Immunol. 39, 596–609 (2018). 30060805 10.1016/j.it.2018.05.005 PMC6154790

[17] D. K. Das, Y. Feng, R. J. Mallis, X. Li, D. B. Keskin, R. E. Hussey, S. K. Brady, J. H. Wang, G. Wagner, E. L. Reinherz, M. J. Lang, Force-dependent transition in the T-cell receptor β-subunit allosterically regulates peptide discrimination and pMHC bond lifetime. Proc. Natl. Acad. Sci. U.S.A. 112, 1517–1522 (2015). 25605925 10.1073/pnas.1424829112 PMC4321250

[18] S. T. Kim, K. Takeuchi, Z.-Y. Sun, M. Touma, C. E. Castro, A. Fahmy, M. J. Lang, G. Wagner, E. L. Reinherz, The alphabeta T cell receptor is an anisotropic mechanosensor. J. Biol. Chem. 284, 31028–31037 (2009). 19755427 10.1074/jbc.M109.052712 PMC2781503

[19] D. K. Das, R. J. Mallis, J. S. Duke-Cohan, R. E. Hussey, P. W. Tetteh, M. Hilton, G. Wagner, M. J. Lang, E. L. Reinherz, Pre-T Cell Receptors (Pre-TCRs) leverage Vβ Complementarity Determining Regions (CDRs) and hydrophobic patch in mechanosensing thymic self-ligands. J. Biol. Chem. 291, 25292–25305 (2016). 27707880 10.1074/jbc.M116.752865 PMC5207233

[20] K. N. Brazin, R. J. Mallis, A. Boeszoermenyi, Y. Feng, A. Yoshizawa, P. A. Reche, P. Kaur, K. Bi, R. E. Hussey, J. S. Duke-Cohan, L. Song, G. Wagner, H. Arthanari, M. J. Lang, E. L. Reinherz, The T cell antigen receptor α transmembrane domain coordinates triggering through regulation of bilayer immersion and CD3 subunit associations. Immunity 49, 829–841.e6 (2018). 30389415 10.1016/j.immuni.2018.09.007 PMC6249037

[21] D. Banik, M. Hamidinia, J. Brzostek, L. Wu, H. M. Stephens, P. A. MacAry, E. L. Reinherz, N. R. J. Gascoigne, M. J. Lang, Single molecule force spectroscopy reveals distinctions in key biophysical parameters of αβ T-cell receptors compared with chimeric antigen receptors directed at the same ligand. J. Phys. Chem. Lett. 12, 7566–7573 (2021). 34347491 10.1021/acs.jpclett.1c02240 PMC9082930

[22] B. Liu, W. Chen, B. D. Evavold, C. Zhu, Accumulation of dynamic catch bonds between TCR and agonist peptide-MHC triggers T cell signaling. Cell 157, 357–368 (2014). 24725404 10.1016/j.cell.2014.02.053 PMC4123688

[23] X. Zhao, E. M. Kolawole, W. Chan, Y. Feng, X. Yang, M. H. Gee, K. M. Jude, L. V. Sibener, P. M. Fordyce, R. N. Germain, B. D. Evavold, K. C. Garcia, Tuning T cell receptor sensitivity through catch bond engineering. Science 376, eabl5282 (2022). 35389803 10.1126/science.abl5282 PMC9513562

[24] P. Wu, T. Zhang, B. Liu, P. Fei, L. Cui, R. Qin, H. Zhu, D. Yao, R. J. Martinez, W. Hu, C. An, Y. Zhang, J. Liu, J. Shi, J. Fan, W. Yin, J. Sun, C. Zhou, X. Zeng, C. Xu, J. Wang, B. D. Evavold, C. Zhu, W. Chen, J. Lou, Mechano-regulation of Peptide-MHC class I conformations determines TCR antigen recognition. Mol. Cell 73, 1015–1027.e7 (2019). 30711376 10.1016/j.molcel.2018.12.018 PMC6408234

[25] W. Hwang, R. J. Mallis, M. J. Lang, E. L. Reinherz, The αβTCR mechanosensor exploits dynamic ectodomain allostery to optimize its ligand recognition site. Proc. Natl. Acad. Sci. U.S.A. 117, 21336–21345 (2020). 32796106 10.1073/pnas.2005899117 PMC7474670

[26] J. Gohring, F. Kellner, L. Schrangl, R. Platzer, E. Klotzsch, H. Stockinger, J. B. Huppa, G. J. Schutz, Temporal analysis of T-cell receptor-imposed forces via quantitative single molecule FRET measurements. Nat. Commun. 12, 2502 (2021). 33947864 10.1038/s41467-021-22775-z PMC8096839

[27] J. Pettmann, L. Awada, B. Rozycki, A. Huhn, S. Faour, M. Kutuzov, L. Limozin, T. R. Weikl, P. A. van der Merwe, P. Robert, O. Dushek, Mechanical forces impair antigen discrimination by reducing differences in T-cell receptor/peptide-MHC off-rates. EMBO J. 42, e111841 (2023). 36484367 10.15252/embj.2022111841 PMC10068313

[28] A. C. Chang-Gonzalez, R. J. Mallis, M. J. Lang, E. L. Reinherz, W. Hwang, Asymmetric framework motion of TCRαβ controls load-dependent peptide discrimination. eLife 13, e91881 (2024). 38167271 10.7554/eLife.91881 PMC10869138

[29] N. Jeffreys, J. M. Brockman, Y. Zhai, D. E. Ingber, D. J. Mooney, Mechanical forces amplify TCR mechanotransduction in T cell activation and function. Appl. Phys. Rev. 11, 011304 (2024). 38434676 10.1063/5.0166848 PMC10848667

[30] T. N. Schumacher, R. D. Schreiber, Neoantigens in cancer immunotherapy. Science 348, 69–74 (2015). 25838375 10.1126/science.aaa4971

[31] C. Puig-Saus, B. Sennino, S. Peng, C. L. Wang, Z. Pan, B. Yuen, B. Purandare, D. An, B. B. Quach, D. Nguyen, H. Xia, S. Jilani, K. Shao, C. McHugh, J. Greer, P. Peabody, S. Nayak, J. Hoover, S. Said, K. Jacoby, O. Dalmas, S. P. Foy, A. Conroy, M. C. Yi, C. Shieh, W. Lu, K. Heeringa, Y. Ma, S. Chizari, M. J. Pilling, M. Ting, R. Tunuguntla, S. Sandoval, R. Moot, T. Hunter, S. Zhao, J. D. Saco, I. Perez-Garcilazo, E. Medina, A. Vega-Crespo, I. Baselga-Carretero, G. Abril-Rodriguez, G. Cherry, D. J. Wong, J. Hundal, B. Chmielowski, D. E. Speiser, M. T. Bethune, X. R. Bao, A. Gros, O. L. Griffith, M. Griffith, J. R. Heath, A. Franzusoff, S. J. Mandl, A. Ribas, Neoantigen-targeted CD8^+^ T cell responses with PD-1 blockade therapy. Nature 615, 697–704 (2023). 36890230 10.1038/s41586-023-05787-1 PMC10441586

[32] K. Ioannidou, P. Baumgaertner, P. O. Gannon, M. F. Speiser, M. Allard, M. Hebeisen, N. Rufer, D. E. Speiser, Heterogeneity assessment of functional T cell avidity. Sci. Rep. 7, 44320 (2017). 28287160 10.1038/srep44320 PMC5347081

[33] P. Dash, A. J. Fiore-Gartland, T. Hertz, G. C. Wang, S. Sharma, A. Souquette, J. C. Crawford, E. B. Clemens, T. H. O. Nguyen, K. Kedzierska, N. L. La Gruta, P. Bradley, P. G. Thomas, Quantifiable predictive features define epitope-specific T cell receptor repertoires. Nature 547, 89–93 (2017). 28636592 10.1038/nature22383 PMC5616171

[34] T. Wu, J. Guan, A. Handel, D. C. Tscharke, J. Sidney, A. Sette, L. M. Wakim, X. Y. X. Sng, P. G. Thomas, N. P. Croft, A. W. Purcell, N. L. La Gruta, Quantification of epitope abundance reveals the effect of direct and cross-presentation on influenza CTL responses. Nat. Commun. 10, 2846 (2019). 31253788 10.1038/s41467-019-10661-8 PMC6599079

[35] A. Babich, S. Li, R. S. O’Connor, M. C. Milone, B. D. Freedman, J. K. Burkhardt, F-actin polymerization and retrograde flow drive sustained PLCγ1 signaling during T cell activation. J. Cell Biol. 197, 775–787 (2012). 22665519 10.1083/jcb.201201018 PMC3373411

[36] M. Ebisuya, K. Kondoh, E. Nishida, The duration, magnitude and compartmentalization of ERK MAP kinase activity: Mechanisms for providing signaling specificity. J. Cell Sci. 118, 2997–3002 (2005). 16014377 10.1242/jcs.02505

[37] M. Hamieh, J. Mansilla-Soto, I. Riviere, M. Sadelain, Programming CAR T Cell Tumor Recognition: Tuned antigen sensing and logic gating. Cancer Discov. 13, 829–843 (2023). 36961206 10.1158/2159-8290.CD-23-0101 PMC10068450

[38] J. P. Lauritsen, M. D. Christensen, J. Dietrich, J. Kastrup, N. Ødum, C. Geisler, Two distinct pathways exist for down-regulation of the TCR. J. Immunol. 161, 260–267 (1998). 9647232

[39] L. E. H. van der Donk, L. S. Ates, J. van der Spek, L. M. Tukker, T. B. H. Geijtenbeek, J. W. J. van Heijst, Separate signaling events control TCR downregulation and T cell activation in primary human T cells. Immun. Inflamm. Dis. 9, 223–238 (2021). 33350598 10.1002/iid3.383 PMC7860602

[40] R. J. Mallis, J. S. Duke-Cohan, D. K. Das, A. Akitsu, A. M. Luoma, D. Banik, H. M. Stephens, P. W. Tetteh, C. D. Castro, S. Krahnke, R. E. Hussey, B. Lawney, K. N. Brazin, P. A. Reche, W. Hwang, E. J. Adams, M. J. Lang, E. L. Reinherz, Molecular design of the γδT cell receptor ectodomain encodes biologically fit ligand recognition in the absence of mechanosensing. Proc. Natl. Acad. Sci. U.S.A. 118, e2023050118 (2021). 34172580 10.1073/pnas.2023050118 PMC8256041

[41] M. Carrion-Vazquez, A. F. Oberhauser, S. B. Fowler, P. E. Marszalek, S. E. Broedel, J. Clarke, J. M. Fernandez, Mechanical and chemical unfolding of a single protein: A comparison. Proc. Natl. Acad. Sci. U.S.A. 96, 3694–3699 (1999). 10097099 10.1073/pnas.96.7.3694 PMC22356

[42] K. Liu, X. Chen, C. M. Kaiser, Energetic dependencies dictate folding mechanism in a complex protein. Proc. Natl. Acad. Sci. U.S.A. 116, 25641–25648 (2019). 31776255 10.1073/pnas.1914366116 PMC6925980

[43] R. H. Pullen III, S. M. Abel, Catch bonds at T Cell interfaces: Impact of surface reorganization and membrane fluctuations. Biophys. J. 113, 120–131 (2017). 28700910 10.1016/j.bpj.2017.05.023 PMC5510709

[44] R. Ma, A. V. Kellner, V. P. Ma, H. Su, B. R. Deal, J. M. Brockman, K. Salaita, DNA probes that store mechanical information reveal transient piconewton forces applied by T cells. Proc. Natl. Acad. Sci. U.S.A. 116, 16949–16954 (2019). 31391300 10.1073/pnas.1904034116 PMC6708336

[45] J. Hong, C. Ge, P. Jothikumar, Z. Yuan, B. Liu, K. Bai, K. Li, W. Rittase, M. Shinzawa, Y. Zhang, A. Palin, P. Love, X. Yu, K. Salaita, B. D. Evavold, A. Singer, C. Zhu, A TCR mechanotransduction signaling loop induces negative selection in the thymus. Nat. Immunol. 19, 1379–1390 (2018). 30420628 10.1038/s41590-018-0259-z PMC6452639

[46] G. J. Kersh, E. N. Kersh, D. H. Fremont, P. M. Allen, High- and low-potency ligands with similar affinities for the TCR: The importance of kinetics in TCR signaling. Immunity 9, 817–826 (1998). 9881972 10.1016/s1074-7613(00)80647-0

[47] Y. H. Ding, B. M. Baker, D. N. Garboczi, W. E. Biddison, D. C. Wiley, Four A6-TCR/peptide/HLA-A2 structures that generate very different T cell signals are nearly identical. Immunity 11, 45–56 (1999). 10435578 10.1016/s1074-7613(00)80080-1

[48] D. J. Nieves, E. Pandzic, S. D. Gunasinghe, J. Goyette, D. M. Owen, J. Justin Gooding, K. Gaus, The T cell receptor displays lateral signal propagation involving non-engaged receptors. Nanoscale 14, 3513–3526 (2022). 35171177 10.1039/d1nr05855j

[49] M. P. Damasio, J. M. Marchingo, L. Spinelli, J. L. Hukelmann, D. A. Cantrell, A. J. M. Howden, Extracellular signal-regulated kinase (ERK) pathway control of CD8^+^ T cell differentiation. Biochem. J. 478, 79–98 (2021). 33305809 10.1042/BCJ20200661 PMC7813476

[50] S. Deswal, A. Meyer, G. J. Fiala, A. E. Eisenhardt, L. C. Schmitt, M. Salek, T. Brummer, O. Acuto, W. W. Schamel, Kidins220/ARMS associates with B-Raf and the TCR, promoting sustained Erk signaling in T cells. J. Immunol. 190, 1927–1935 (2013). 23359496 10.4049/jimmunol.1200653

[51] M. Krajnik, M. Schafer, P. Sobanski, J. Kowalewski, E. Bloch-Boguslawska, Z. Zylicz, S. A. Mousa, Enkephalin, its precursor, processing enzymes, and receptor as part of a local opioid network throughout the respiratory system of lung cancer patients. Hum. Pathol. 41, 632–642 (2010). 20040394 10.1016/j.humpath.2009.08.025

[52] T. R. Cox, J. T. Erler, Remodeling and homeostasis of the extracellular matrix: Implications for fibrotic diseases and cancer. Dis. Model. Mech. 4, 165–178 (2011). 21324931 10.1242/dmm.004077 PMC3046088

[53] H. Du, J. M. Bartleson, S. Butenko, V. Alonso, W. F. Liu, D. A. Winer, M. J. Butte, Tuning immunity through tissue mechanotransduction. Nat. Rev. Immunol. 23, 174–188 (2023). 35974148 10.1038/s41577-022-00761-w PMC9379893

[54] J. Huang, L. Zhang, D. Wan, L. Zhou, S. Zheng, S. Lin, Y. Qiao, Extracellular matrix and its therapeutic potential for cancer treatment. Signal Transduct. Target. Ther. 6, 153 (2021). 33888679 10.1038/s41392-021-00544-0 PMC8062524

[55] K. L. Cepek, S. K. Shaw, C. M. Parker, G. J. Russell, J. S. Morrow, D. L. Rimm, M. B. Brenner, Adhesion between epithelial cells and T lymphocytes mediated by E-cadherin and the αEβ_7_ integrin. Nature 372, 190–193 (1994). 7969453 10.1038/372190a0

[56] S. A. Migueles, D. M. Nettere, N. V. Gavil, L. T. Wang, S. A. Toulmin, E. P. Kelly, A. J. Ward, S. Lin, S. A. Thompson, B. A. Peterson, C. S. Abdeen, C. R. Sclafani, P. F. Pryal, B. G. Leach, A. K. Ludwig, D. C. Rogan, P. A. Przygonska, A. Cattani, H. Imamichi, A. Sachs, G. Cafri, N. N. Huang, A. Patamawenu, C. J. Liang, C. W. Hallahan, D. M. Kambach, E. X. Han, T. Coupet, J. Chen, S. L. Moir, T. W. Chun, E. E. Coates, J. Ledgerwood, J. Schmidt, M. Taillandier-Coindard, J. Michaux, H. Pak, M. Bassani-Sternberg, N. Frahm, M. J. McElrath, M. Connors, HIV vaccines induce CD8^+^ T cells with low antigen receptor sensitivity. Science 382, 1270–1276 (2023). 38096385 10.1126/science.adg0514

[57] S. Sengupta, N. L. Board, F. Wu, M. Moskovljevic, J. Douglass, J. Zhang, B. R. Reinhold, J. Duke-Cohan, J. Yu, M. C. Reed, Y. Tabdili, A. Azurmendi, E. J. Fray, H. Zhang, E. H. Hsiue, K. Jenike, Y. C. Ho, S. B. Gabelli, K. W. Kinzler, B. Vogelstein, S. Zhou, J. D. Siliciano, S. Sadegh-Nasseri, E. L. Reinherz, R. F. Siliciano, TCR-mimic bispecific antibodies to target the HIV-1 reservoir. Proc. Natl. Acad. Sci. U.S.A. 119, e2123406119 (2022). 35394875 10.1073/pnas.2123406119 PMC9169739

[58] O. Schwartz, V. Maréchal, S. Le Gall, F. Lemonnier, J. M. Heard, Endocytosis of major histocompatibility complex class I molecules is induced by the HIV-1 Nef protein. Nat. Med. 2, 338–342 (1996). 8612235 10.1038/nm0396-338

[59] A. Straub, S. Grassmann, S. Jarosch, L. Richter, P. Hilgendorf, M. Hammel, K. I. Wagner, V. R. Buchholz, K. Schober, D. H. Busch, Recruitment of epitope-specific T cell clones with a low-avidity threshold supports efficacy against mutational escape upon re-infection. Immunity 56, 1269–1284.e6 (2023). 37164014 10.1016/j.immuni.2023.04.010

[60] A. Yoshizawa, K. Bi, D. B. Keskin, G. Zhang, B. Reinhold, E. L. Reinherz, TCR-pMHC encounter differentially regulates transcriptomes of tissue-resident CD8 T cells. Eur. J. Immunol. 48, 128–150 (2018). 28872670 10.1002/eji.201747174 PMC6531858

[61] K. G. Anderson, K. Mayer-Barber, H. Sung, L. Beura, B. R. James, J. J. Taylor, L. Qunaj, T. S. Griffith, V. Vezys, D. L. Barber, D. Masopust, Intravascular staining for discrimination of vascular and tissue leukocytes. Nat. Protoc. 9, 209–222 (2014). 24385150 10.1038/nprot.2014.005 PMC4428344

[62] E. Kobayashi, E. Mizukoshi, H. Kishi, T. Ozawa, H. Hamana, T. Nagai, H. Nakagawa, A. Jin, S. Kaneko, A. Muraguchi, A new cloning and expression system yields and validates TCRs from blood lymphocytes of patients with cancer within 10 days. Nat. Med. 19, 1542–1546 (2013). 24121927 10.1038/nm.3358

[63] H. Hamana, K. Shitaoka, H. Kishi, T. Ozawa, A. Muraguchi, A novel, rapid and efficient method of cloning functional antigen-specific T-cell receptors from single human and mouse T-cells. Biochem. Biophys. Res. Commun. 474, 709–714 (2016). 27155153 10.1016/j.bbrc.2016.05.015

[64] J. Holst, A. L. Szymczak-Workman, K. M. Vignali, A. R. Burton, C. J. Workman, D. A. Vignali, Generation of T-cell receptor retrogenic mice. Nat. Protoc. 1, 406–417 (2006). 17406263 10.1038/nprot.2006.61

[65] J. Liu, A. G. Tse, H. C. Chang, J. Liu, J. Wang, R. E. Hussey, Y. Chishti, B. Rheinhold, R. Spoerl, S. G. Nathenson, J. C. Sacchettini, E. L. Reinherz, Crystallization of a deglycosylated T cell receptor (TCR) complexed with an anti-TCR Fab fragment. J. Biol. Chem. 271, 33639–33646 (1996). 8969233 10.1074/jbc.271.52.33639

[66] T. Uematsu, E. Iizasa, N. Kobayashi, H. Yoshida, H. Hara, Loss of CARD9-mediated innate activation attenuates severe influenza pneumonia without compromising host viral immunity. Sci. Rep. 5, 17577 (2015). 26627732 10.1038/srep17577 PMC4667252

[67] A. Dobin, C. A. Davis, F. Schlesinger, J. Drenkow, C. Zaleski, S. Jha, P. Batut, M. Chaisson, T. R. Gingeras, STAR: Ultrafast universal RNA-seq aligner. Bioinformatics 29, 15–21 (2013). 23104886 10.1093/bioinformatics/bts635 PMC3530905

[68] M. Cornwell, M. Vangala, L. Taing, Z. Herbert, J. Koster, B. Li, H. Sun, T. Li, J. Zhang, X. Qiu, M. Pun, R. Jeselsohn, M. Brown, X. S. Liu, H. W. Long, VIPER: Visualization Pipeline for RNA-seq, a Snakemake workflow for efficient and complete RNA-seq analysis. BMC Bioinformatics 19, 135 (2018). 29649993 10.1186/s12859-018-2139-9 PMC5897949

[69] M. I. Love, W. Huber, S. Anders, Moderated estimation of fold change and dispersion for RNA-seq data with DESeq2. Genome Biol. 15, 550 (2014). 25516281 10.1186/s13059-014-0550-8 PMC4302049

[70] T. Wu, E. Hu, S. Xu, M. Chen, P. Guo, Z. Dai, T. Feng, L. Zhou, W. Tang, L. Zhan, X. Fu, S. Liu, X. Bo, G. Yu, clusterProfiler 4.0: A universal enrichment tool for interpreting omics data. Innovation (Camb) 2, 100141 (2021). 34557778 10.1016/j.xinn.2021.100141 PMC8454663

