# Supplementary material for: Parsing digital or analog TCR performance through piconewton forces
Source: Sci Adv. 2024 Aug 14;10(33):eado4313. doi: 10.1126/sciadv.ado4313 (PMC11323890; doi:10.1126/sciadv.ado4313)
Supplement: Supplementary file 1 — Figs. S1 to S10 Data S2, S4, and S8 Legends for movies S1 to S3 Legends for data S1 to S8 [file sciadv.ado4313_sm.pdf]

Science Advances
AAAS

Supplementary Materials for

Parsing digital or analog TCR performance through piconewton forces

Aoi Akitsu *et al.*

Corresponding author: Matthew J. Lang, matt.lang@vanderbilt.edu;
Ellis L. Reinherz, ellis_reinherz@dfci.harvard.edu

Sci. Adv. **10**, eado4313 (2024)
DOI: 10.1126/sciadv.ado4313

The PDF file includes:

Figs. S1 to S10
Data S2, S4, and S8
Legends for movies S1 to S3
Legends for data S1 to S8

Other Supplementary Material for this manuscript includes the following:

Movies S1 to S3
Data S1 to S8

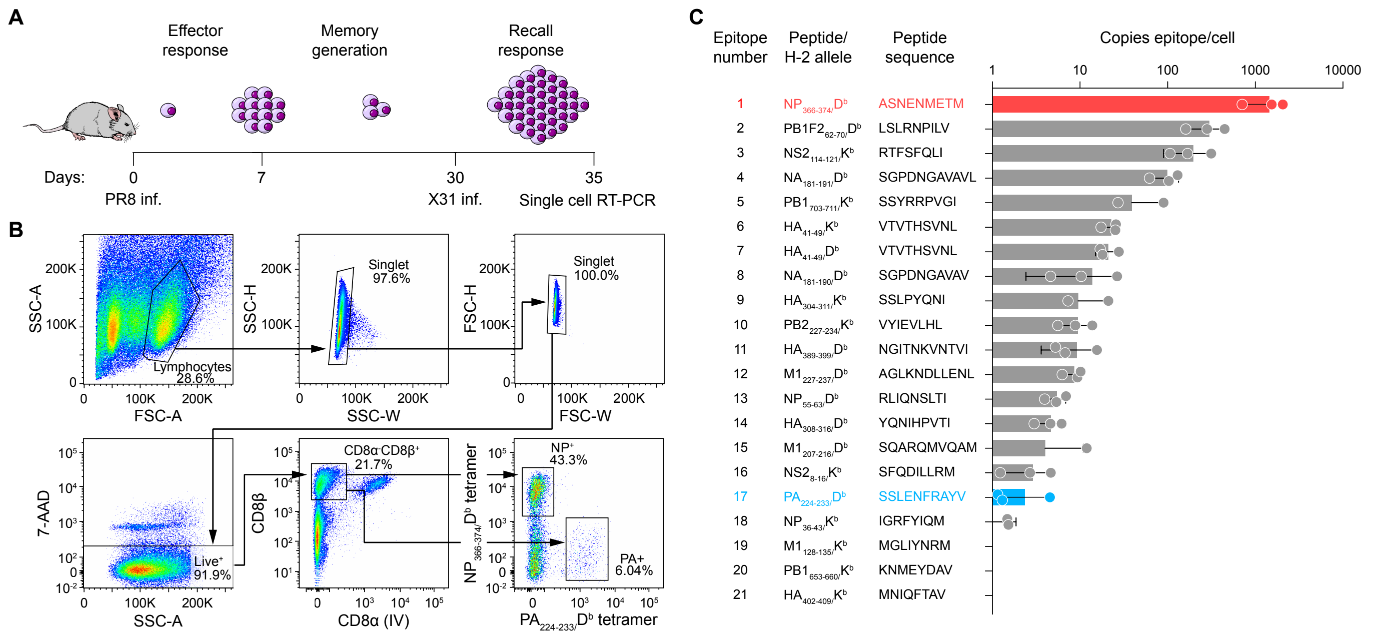
A
Effector response
Memory generation
Recall response
Days: 0 7 30 35
PR8 inf.
X31 inf.
Single cell RT-PCR
B
SSC-A
FSC-A
Lymphocytes 28.6%
SSC-H
SSC-W
Singlet 97.6%
FSC-H
FSC-W
Singlet 100.0%
7-AAD
SSC-A
Live+ 91.9%
CD8β
CD8α (IV)
CD8α-CD8β+ 21.7%
NP366-374/Db tetramer
PA224-233/Db tetramer
NP+ 43.3%
PA+ 6.04%
C
Epitope number
Peptide/ H-2 allele
Peptide sequence
Copies epitope/cell
1 10 100 1000 10000
1 NP366-374/Db ASNENMETM
2 PB1F262-70/Db LSLRNPILV
3 NS2114-121/Kb RTFSFQLI
4 NA181-191/Db SGPDNGAVAVL
5 PB1703-711/Kb SSYRRPVGI
6 HA41-49/Kb VTVTHSVNL
7 HA41-49/Db VTVTHSVNL
8 NA181-190/Db SGPDNGAVAV
9 HA304-311/Kb SSLPYQNI
10 PB2227-234/Kb VYIEVLHL
11 HA389-399/Db NGITNKVNTVI
12 M1227-237/Db AGLKNDLLENL
13 NP55-63/Db RLIQNSLTI
14 HA308-316/Db YQNIHPVTI
15 M1207-216/Db SQARQMVQAM
16 NS28-16/Kb SFQDILLRM
17 PA224-233/Db SSLENFRAYV
18 NP36-43/Kb IGRFYIQM
19 M1128-135/Kb MGLIYNRM
20 PB1653-660/Kb KNMEYDAV
21 HA402-409/Kb MNIQFTAV

Fig. S1. Schematic of cell isolation for single-cell RT-PCR and analysis of $NP_{366-374}/D^b$- and $PA_{224-233}/D^b$- specific TCR directed at distinct peptides with divergent copy numbers after IAV infection.

(A) Workflow to clone $NP_{366-374}/D^b$- and $PA_{224-233}/D^b$- specific TCRs derived from lung resident $CD8^+$ T cells. T cells were isolated 5 days post-recall by cell sorting and single cell RT-PCR. (**B**) Sorting strategy to isolate $NP_{366-374}/D^b$- and $PA_{224-233}/D^b$- specific T cells. Lymphocytes were selected based on FSC-A and SSC-A, then singlets were gated. Live tissue-resident cells were then gated using 7-AAD^-, $CD8\beta^+$, intravascular stained $CD8\alpha^-$, while $NP_{366-374}/D_b$- and $PA_{224-233}/D_b$- specific T cells were simultaneously sorted as $NP_{366-374}/D_b$-$tetramer^+$ and $PA_{224-233}/D_b$-$tetramer^+$ cells, respectively. **(C)** Copy number of 21 peptide/H-2 MHC complexes on LET1 cells infected with Influenza A/PR/8/34 virus (PR8) based upon data adapted from Wu et al. *Nature communications* (2019). The pMHCs were isolated from infected LET1 cells, and then the peptides were eluted from their immunoaffinity-purified K^b and D^b MHCI molecules and analyzed by LC-MS as described. Results from LET1 are summarized, with $NP_{366-374}/D^b$- and $PA_{224-233}/D^b$ peptide copy numbers adapted from that publication.

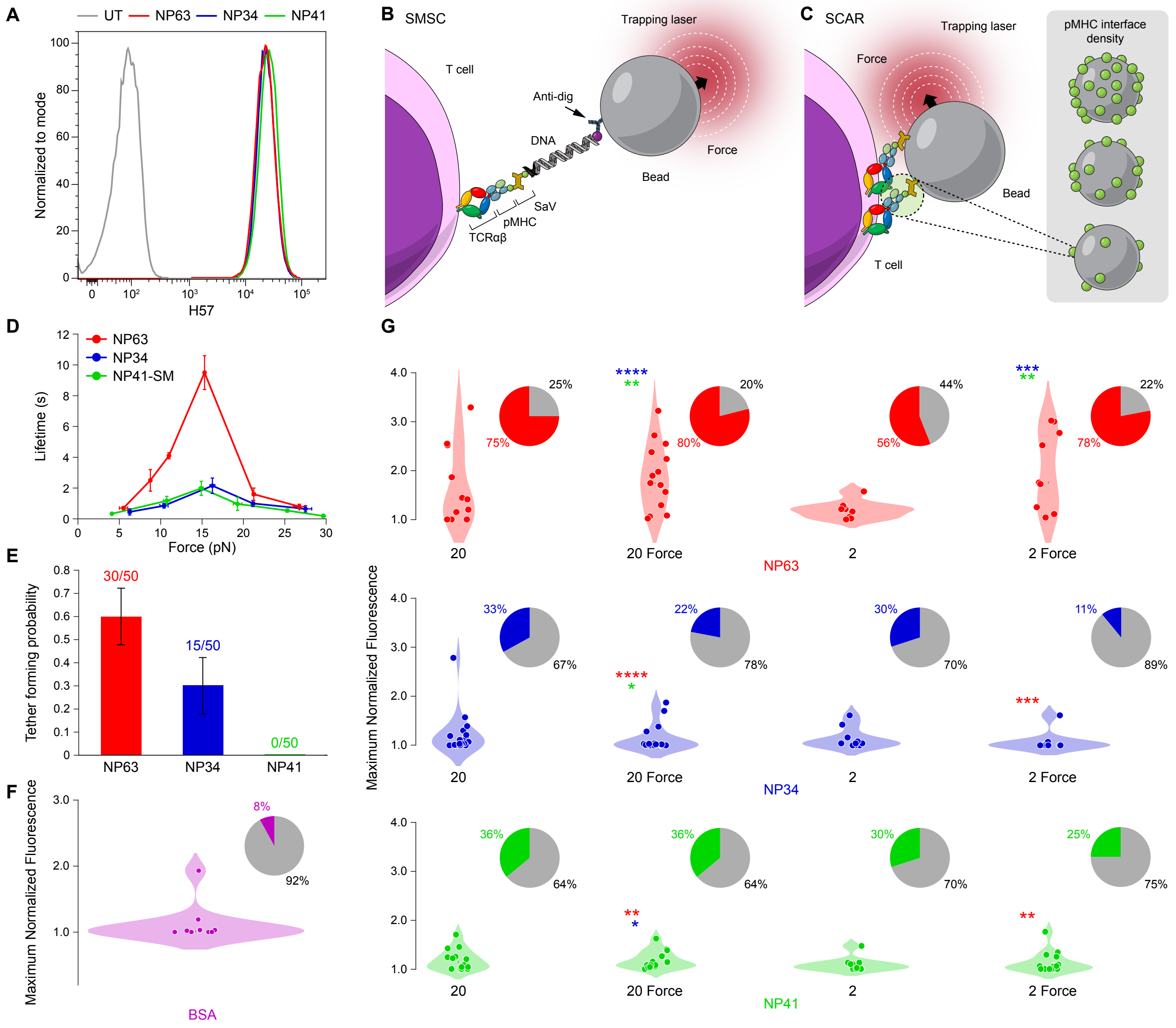
A
UT
NP63
NP34
NP41
Normalized to mode
H57
B
SMSC
T cell
Trapping laser
Anti-dig
DNA
Force
Bead
SaV
pMHC
TCRαβ
C
SCAR
Trapping laser
Force
Bead
T cell
pMHC interface density
D
NP63
NP34
NP41-SM
Lifetime (s)
Force (pN)
E
Tether forming probability
30/50
15/50
0/50
NP63
NP34
NP41
F
Maximum Normalized Fluorescence
8%
92%
BSA
G
Maximum Normalized Fluorescence
25%
75%
20%
80%
44%
56%
22%
78%
20
20 Force
2
2 Force
NP63
33%
67%
22%
78%
30%
70%
11%
89%
NP34
36%
64%
36%
64%
30%
70%
25%
75%
NP41

Fig. S2. The greater activation under force of the digital NP63 TCR relative to analogue NP34 and NP41.

(A) TCRβ expression on CD8αβ^{+} TCR^{-} BW5147 cell line transduced with NP34, NP41, and NP63 TCRαβ after cell sorting to match TCR expression as defined by H57 anti-Cβ mAb. (**B**) Single molecule single cell (SMSC) assay design for optical tweezer experiments. Beads functionalized with DNA tethers terminating in pMHC are actively introduced to the cell surface to initiate bond-formation, then retracted for bond-loading. Movement of the bead relative to the cell is controlled by a piezo stage that translates the cell relative to a stationary trapping laser. (**C**) Single cell activation requirement (SCAR) assay design for optical tweezer experiments. T cells are introduced to pMHC coated beads, with varying densities of an agonist pMHC (NP $_{366\text{-}374}$/D^{b} or PA$_{224\text{-}233}$ /D^{b}), to promote bond formation at the interface. Moving the piezo stage relative to the T cell applies a vectorial force to the system via the optical trap. (**D**) Force vs. lifetime distributions, comparing NP63 and NP34 obtained with SMSC assay to NP41 obtained with SM assay. (**E**) Tether forming probability in the single-molecule single-cell (SMSC) assay under identical bead and coverslip conditions for each clone. At conditions where sufficient tethers are formed between NP $_{366\text{-}374}$/D^{b} pMHC and NP63 or NP34 cells, no tethers are formed in the case of NP41. (**F**) SCAR assay conducted with BSA beads as a control for the pMHC experiments. This control was conducted on the PA59 cell line, a digital responder. The bead's surface was saturated with BSA, which should not trigger the T cells upon contact. Each dot in the violin plot corresponds to the maximum normalized calcium flux of a single cell. The normalized calcium flux is represented as a ratio between the maximum fluorescence intensity (I_{max}) and the initial fluorescence intensity (I_0). Pie chart representation is defined in panel G. **(G)** SCAR assay used to measure the calcium flux in NP34-, NP41-, and NP63- BW cells using either 2 or 20 interfacial copy number of NP$_{366\text{-}}$

$_{374}$/D^{b}, conducted on a high-resolution dual fluorescence -OT microscope (see SCAR methods for more details). Experiments were conducted with and without external force application as indicated. Each dot in the violin plot corresponds to the maximum normalized calcium flux of a single cell in that data bin. The cutoff between non-triggering and triggering cells is typically a maximum normalized fluorescence of 1.1, although a few exceptions were made due to profile shape. Bulges in the violin plot below 1.1 intensity are indicative of a high non-triggering population in the data set. A more distributed population is an indication that the cells are triggering at different intensities. The pie chart shows the triggering percentage where the solid color triggered and grey color is non-triggered. Significance between cells of the same bin category are color coded to match their respective cells. For all data statistics, ****P<0.0001, ***P<0.001, **P<0.01, *P<0.05. P values for violin plots were calculated by the Kruskal-Wallis test.

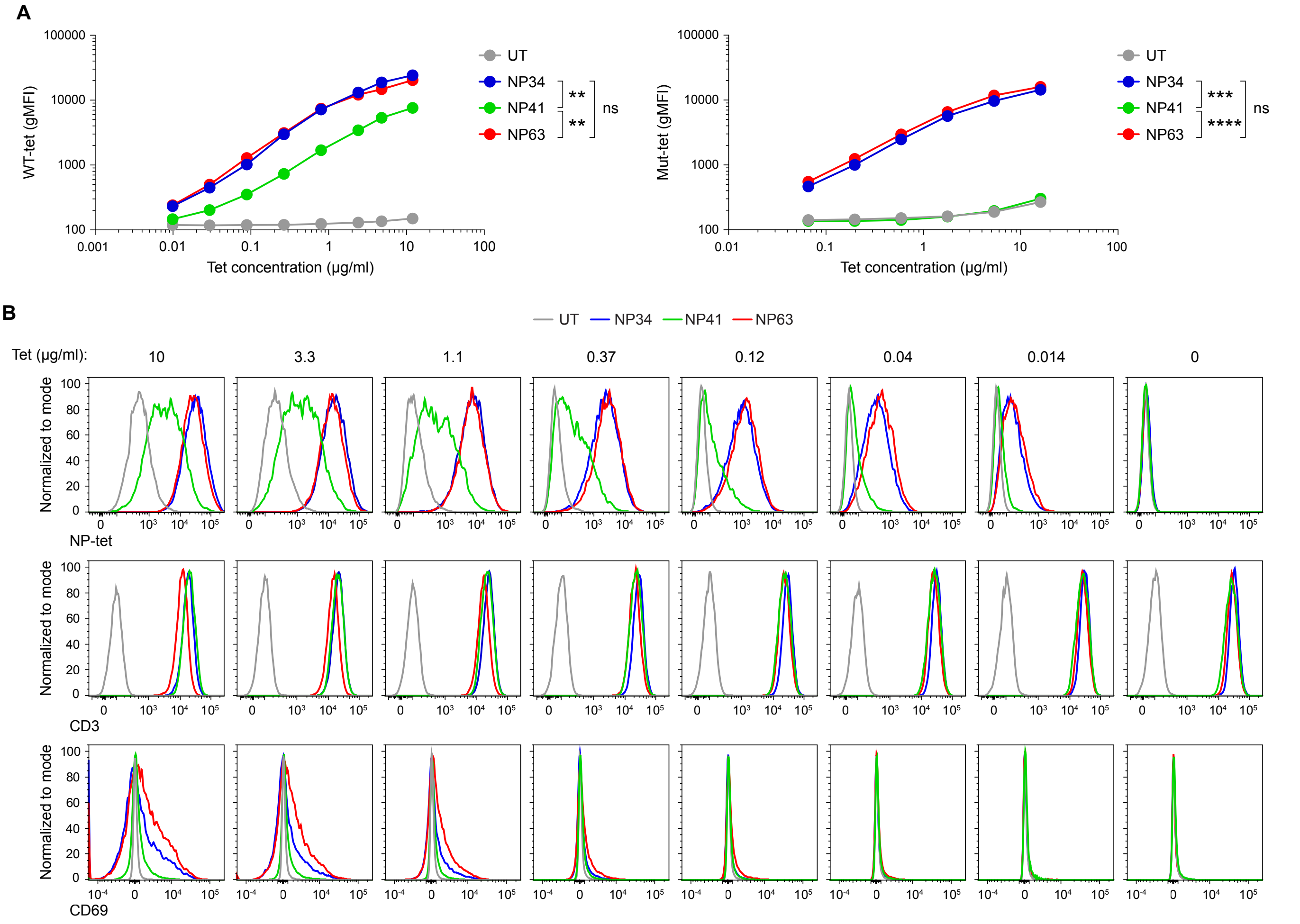

A
WT-tet (gMFI)
Tet concentration (μg/ml)
UT
NP34
NP41
NP63
**
**
ns
Mut-tet (gMFI)
Tet concentration (μg/ml)
***
****
ns
B
UT NP34 NP41 NP63
Tet (μg/ml):
10
3.3
1.1
0.37
0.12
0.04
0.014
0
Normalized to mode
NP-tet
CD3
CD69

Fig. S3. Superior activation ability of T cells expressing digital NP63 TCR in response to tetramer stimulation *in vitro*.

(A) Tetramer binding of $NP_{366\text{-}374}$ /D^b WT-tetramer (left) and CD8BS-mutant tetramer (right) to NP34-, 41-, and 63-BW cell lines and BW UT, cells untransduced with a NP-specific TCR. Tetramer treatment was performed at 20 °C for 30 minutes. Data are representative of two independent experiments. ****P <0.0001, **P<0.01; ns, not significant. P values were calculated by linear regression. **(B)** Histogram of tetramer (top), anti-CD3 (middle), and anti-CD69 binding (bottom) for NP34 (blue)-, 41 (green)-, and 63 (red)-BW cell lines after stimulation with indicated concentration of $NP_{366\text{-}374}$ /D^b WT-tetramer at 37 °C overnight, as shown in Fig. 1F, I and J, respectively. Data are representative of three replicates in two independent experiments.

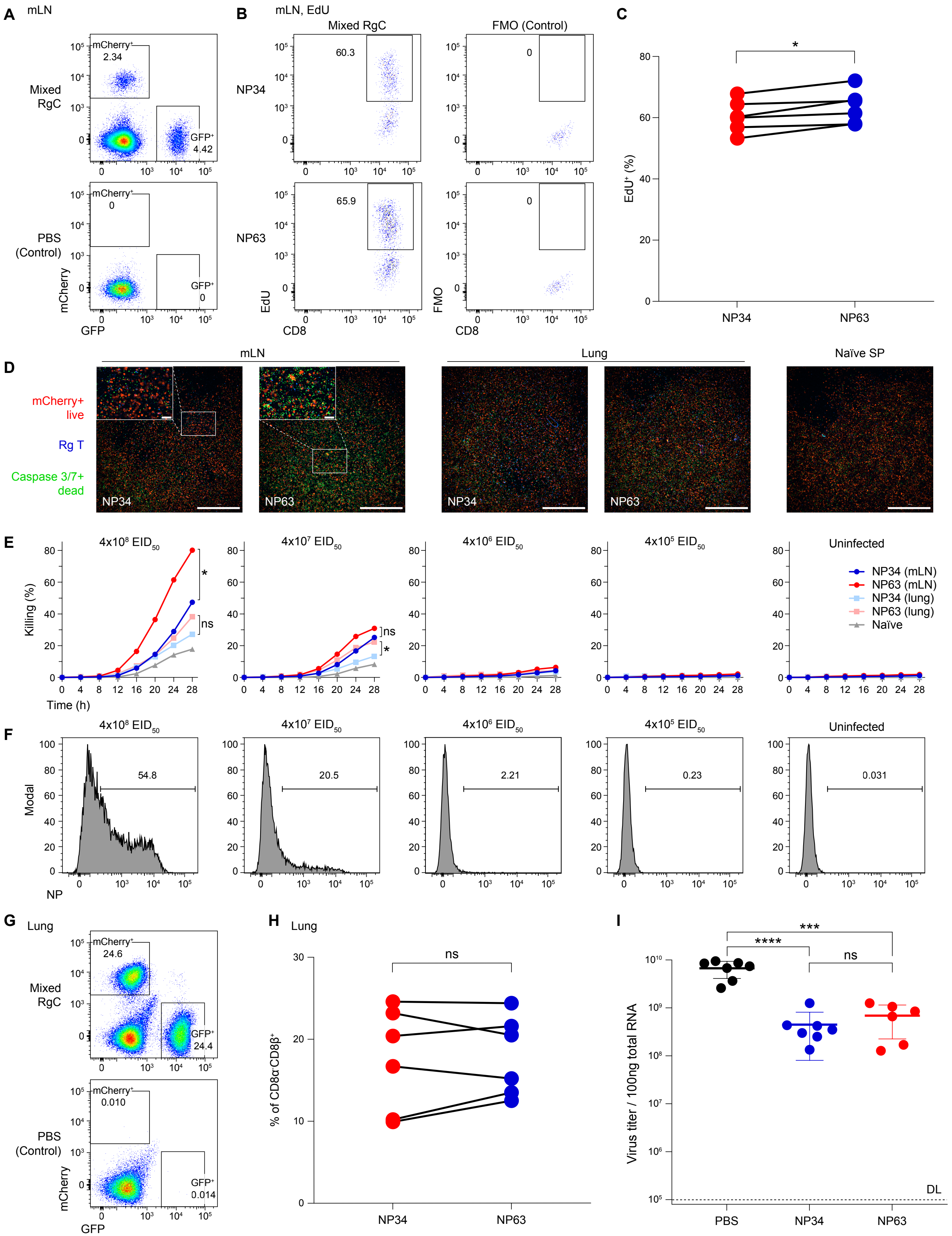
A
mLN
Mixed RgC
PBS (Control)
mCherry⁺
2.34
GFP⁺
4.42
0
mCherry
GFP
B
mLN, EdU
Mixed RgC
FMO (Control)
NP34
NP63
60.3
65.9
EdU
FMO
CD8
C
EdU⁺ (%)
*
NP34
NP63
D
mLN
Lung
Naïve SP
mCherry+ live
Rg T
Caspase 3/7+ dead
E
4x10⁸ EID50
4x10⁷ EID50
4x10⁶ EID50
4x10⁵ EID50
Uninfected
Killing (%)
Time (h)
ns
NP34 (mLN)
NP63 (mLN)
NP34 (lung)
NP63 (lung)
Naïve
F
Modal
NP
54.8
20.5
2.21
0.23
0.031
G
Lung
24.6
24.4
0.010
0.014
H
% of CD8α⁻CD8β⁺
I
Virus titer / 100ng total RNA
****
***
DL
PBS

Fig. S4. NP34 and NP63 Rg T cell activation *in vivo* after IAV infection.

(A) Representative FACS plots of NP34 (mCherry$^+$) and NP63 (GFP$^+$) T cells in mLN of mixed RgC mice 7 days after PR8 infection shown in Fig. 1K. Data were derived after first gating on CD8b$^+$ cells. Control mice were injected with PBS without Rg T cells when the RgC mice were generated. (**B, C**) EdU incorporation in NP34 and NP63 T cells. Representative FACS plots (**B**) and quantification (**C**) are shown. (**D**) Original images of mLN Rg T cell- (top) and lung Rg T cell (bottom)-mediated killing of LET1 cells infected with 4x10^8 EID_{50} PR8 at 20 hours, as shown in Fig. 1M. Naïve splenic T cells were used as control (Naïve SP) of uninfected B6 mice (bottom right). Rg T cells were sorted from six pooled RgC mice that received a single RgT cell type (dpi 7) and then cultured on infected LET1 cells. LET1 cells are visualized by transduced mCherry, apoptotic cells are visualized in green using Caspase-3/7 Green ReadyProbes, and Rg T cells are stained in blue with Cell Proliferation Dye eFluor 450. mLN NP63 Rg T cells show a significant killing ability indicated by increased green apoptotic signals and loss of mCherry$^+$ LET1 cells. Lower magnitude but nonetheless superior killing is also evident for lung NP63 relative to NP34 T cells. Scale bars in main figure panels indicate 1000 μm. Upper left windows are zoomed in views shown in Fig.1M relative to the larger image with the squares outlined in white dotted lines indicating their position in the original images. Scale bars in white windows indicate 100 μm. (**E)** Time-course of T cell-mediated killing of LET1 cells infected PR8 with indicated doses. mLN and lung Rg T cells were derived from RgC mice adoptively transferred with a single RgT type (dpi 7). (**F**) Intracellular NP protein expression in LET1 cells infected with the indicated dose of PR8 and then analyzed by flow cytometry. (**G, H**) Representative FACS plots (**G**) and the quantification (**H**) of NP34 (mCherry$^+$) and NP63 (GFP$^+$) T cells in lungs of mixed RgC mice 7 days after PR8 infection. Intraparenchymal lung T cells shown are identified by lack of anti-CD8α staining after

in vivo intravenous staining in conjunction with anti-CD8β reactivity following *in vitro* staining. (**I**) Viral titer in lungs of single NP34- and 63-RgC mice (dpi 7). For **A-C, G, and H**, data are representative of four independent experiments. For **D-F, and I**, data are representative of two independent experiments and are shown as means ± SDs of six to eight mice. For all data with statistics, ****P <0.0001, ***P <0.001, *P<0.05; ns, not significant. P values were calculated by comparing slopes using linear regression analysis (**E**), paired t-test (**C and H**), or unpaired t-test (**I**).

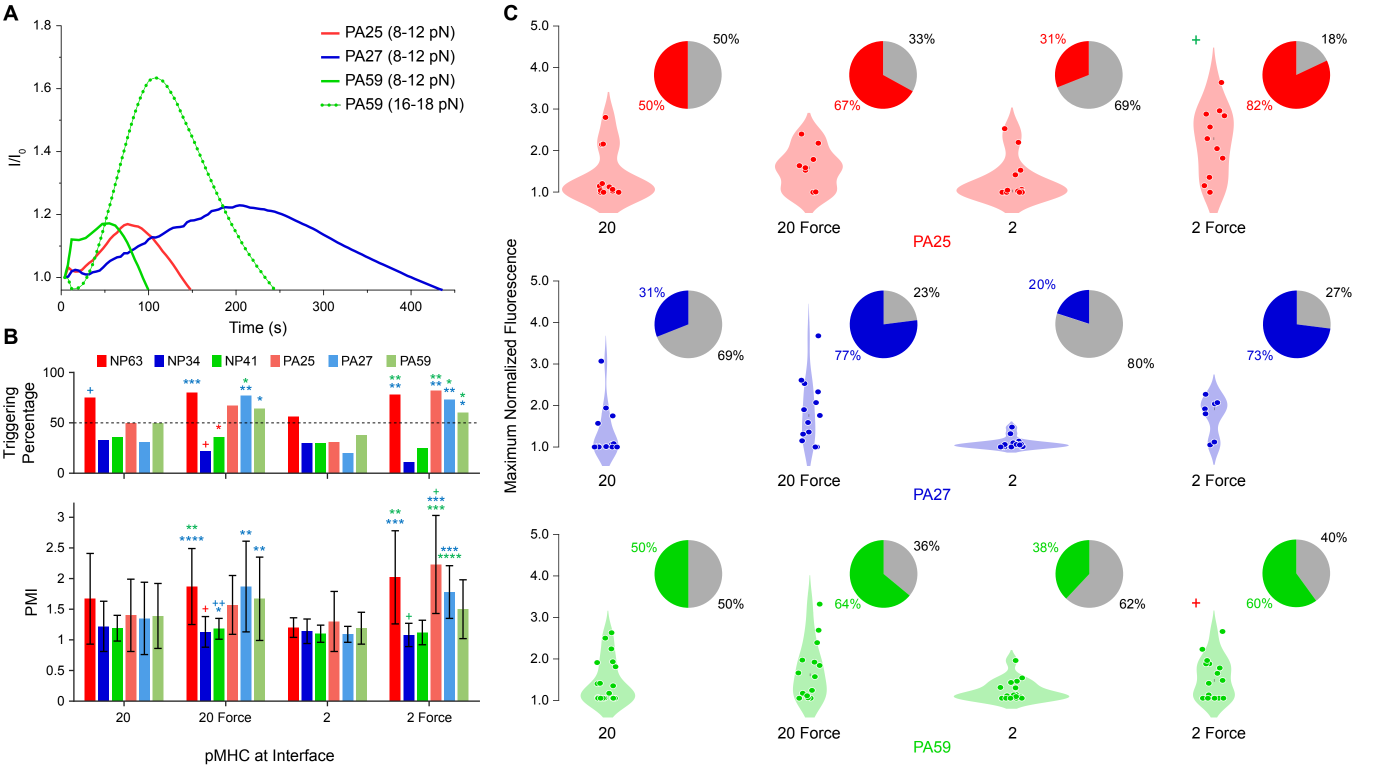

A
PA25 (8-12 pN)
PA27 (8-12 pN)
PA59 (8-12 pN)
PA59 (16-18 pN)
I/I0
Time (s)
B
NP63
NP34
NP41
PA25
PA27
PA59
Triggering Percentage
PMI
20
20 Force
2
2 Force
pMHC at Interface
C
Maximum Normalized Fluorescence
PA25
PA27
PA59

Fig. S5. SCAR assay revealing the prolonged calcium flux in PA27 and the strong magnitude in PA59 under higher force.

(A) Time-course of calcium flux signal indicated as the ratio of maximum fluorescence intensity (I_{max}) to the initial fluorescence intensity (I_0) of the Ca^{2+}-sensitive dye for PA25-, PA27-, PA59-BW cells at 8-12 pN (solid lines) and PA59 at 16-18 pN (dotted line). **(B)** Quantification of triggering percentage and PMI (Predicted Mean Intensity; average normalized fluorescence for all cells in a bin) for NP34-, NP41-, and NP63- and PA25-, PA27-, and PA59- BW cells with the indicated interfacial number of pMHC with or without force application. The cells were activated with their cognate pMHC $NP_{366-374}/D^b$ and $PA_{224-233}/D^b$, respectively. The dashed line is used to indicate a triggering percentage of 50%. The significance between different cell lines is indicated by different symbols. Significance against the NP lines is represented by a *, while significance against the PA lines is represented by a +. These symbols are also color coded to their respective cells. **(C)** SCAR assay used to measure the calcium flux in PA25-, PA27-, and PA59- BW cells using either 2 or 20 interfacial copy number of $PA_{224-233}/D^b$, conducted on a high-resolution dual fluorescence -OT microscope (see SCAR methods for more details). Experiments were conducted with and without external force application as indicated. Each dot in the violin plot corresponds to the maximum normalized calcium flux of a single cell in that data bin. The normalized calcium flux is represented as a ratio between the maximum fluorescence intensity (I_{max}) and the initial fluorescence intensity (I_0). The cutoff between non-triggering and triggering cells is typically a maximum normalized fluorescence of 1.1, although a few exceptions were made due to profile shape. Bulges in the violin plot below 1.1 intensity are indicative of a high non-triggering population in the data set. A more distributed population is an indication that the cells are triggering at different intensities. The pie chart shows the triggering percentage where the solid color is

triggered and grey color is non-triggered. Significance is represented by a + to match the symbol convention in **B**. These symbols are also color coded to their respective cells. For **B**, data are shown as mean ± SD. For all data statistics, ****P<0.0001, ***P<0.001, **P<0.01, *P<0.05. P values for triggering percentages were calculated by Pearson's chi-squared test. P values for PMIs in **B** and violin plots in **C** were calculated by the Kruskal-Wallis test.

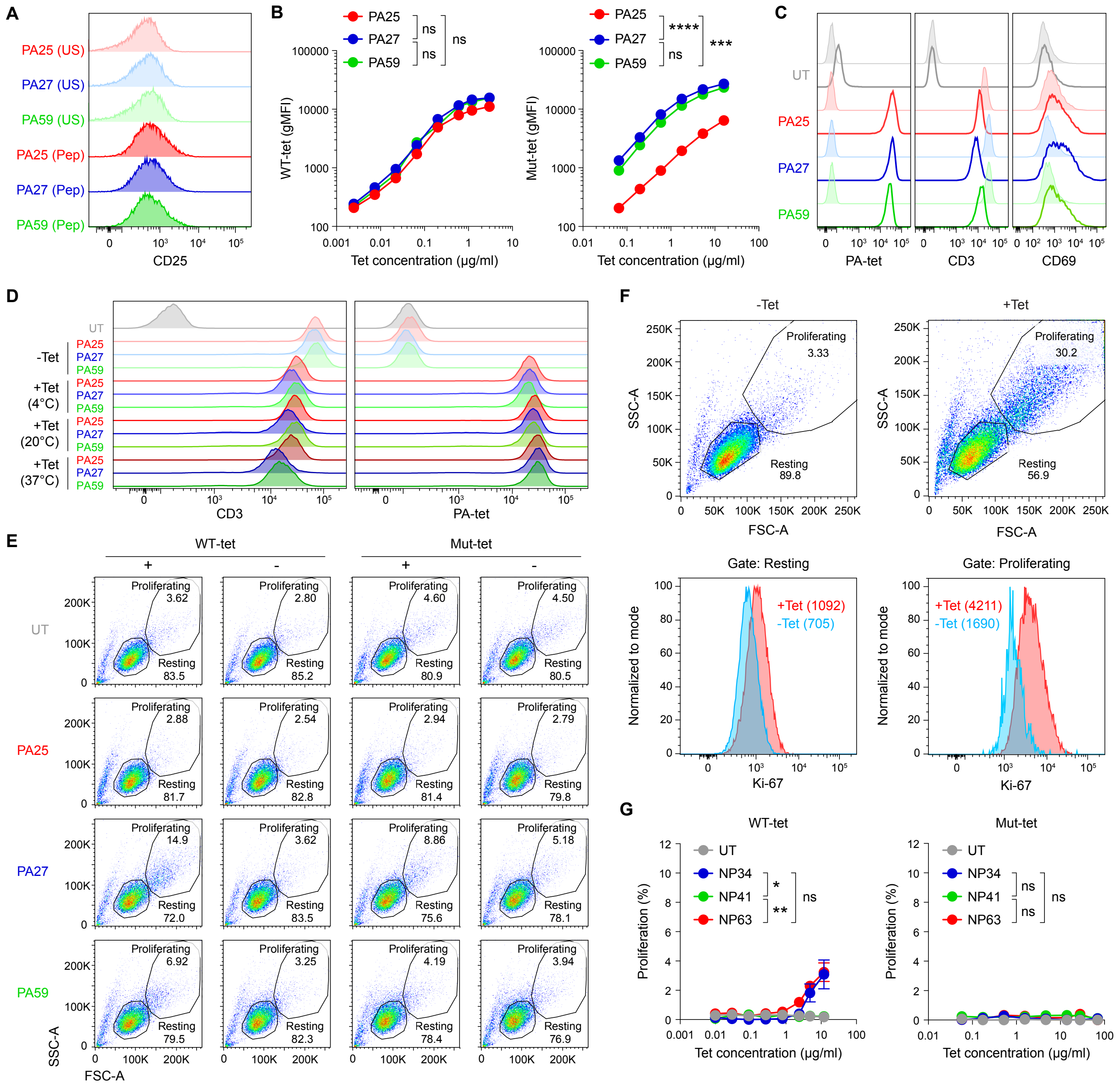
A
PA25 (US)
PA27 (US)
PA59 (US)
PA25 (Pep)
PA27 (Pep)
PA59 (Pep)
CD25
B
PA25
PA27
PA59
ns
ns
ns
WT-tet (gMFI)
Tet concentration (µg/ml)
****
***
Mut-tet (gMFI)
C
UT
PA-tet
CD3
CD69
D
-Tet
+Tet (4°C)
+Tet (20°C)
+Tet (37°C)
E
WT-tet
Mut-tet
Proliferating
Resting
SSC-A
FSC-A
F
-Tet
+Tet
Proliferating 3.33
Resting 89.8
Proliferating 30.2
Resting 56.9
Gate: Resting
Gate: Proliferating
+Tet (1092)
-Tet (705)
+Tet (4211)
-Tet (1690)
Normalized to mode
Ki-67
G
UT
NP34
NP41
NP63
Proliferation (%)

Fig. S6. The greatest proliferation and activation of PA TCR-expressing cells is observed for PA27 after tetramer stimulation *in vitro*.

(**A**) CD25 expression on the indicated TCR-transduced BW cells unstimulated (US) or stimulated with 10μg/ml $PA_{224-233}$ peptide (Pep) overnight. (**B**) Tetramer binding of $PA_{224-233}$ /D^b WT-tetramer (left) and CD8BS mutant-tetramer (right) for the indicated BW cell lines. Tetramer was treated for 30 minutes at 20 °C. (**C**) Representative histograms of PA-tetramer binding (left) as well as CD3 (middle) and CD69 expression (right) for PA25-, 27-, 59-, and untransduced-BW cell lines following overnight stimulation with 10 μg/mL $PA_{224-233}$ /D^b WT-tetramer at 37 °C (bold lines), as shown in Fig. 2F, I, and J, respectively. Shaded histograms represent control culture without tetramer addition. (**D**) CD3 expression (left) and tetramer binding (right) on indicated BW cells 30 minutes after $PA_{224-233}$ /D^b WT-tetramer stimulation at indicated temperatures. (**E)** Representative FACS plots of FSC-A and SSC-A for the indicated BW cells cultured for 1 hour at 37 °C with $PA_{224-233}$ /D^b WT-tetramer (3 μg/mL), its CD8BS-mutant tetramer variant (22 μg/mL), or no tetramer. The cells with larger FSC and SSC are identified as proliferating cells shown in Fig. 2K and L. (**F**) Proliferation molecule Ki67 expression in PA27-BW cells with or without $PA_{224-233}$ /D^b WT-tetramer stimulation (1.2 μg/mL) for 1 hour at 37 °C. Top panels show proliferating and resting cells based on SSC-A and FSC-A scatter used for gating to determine Ki67 levels shown in the bottom row. With values in parentheses indicators gMFI. (**G**) Proliferation of indicated NP-BW cells with $NP_{366-374}$ /D^b WT-tetramer (left) and CD8BS mutant-tetramer (right) stimulation. Data are normalized by subtracting the percentage of proliferating cells without stimulation from the percentage with stimulation. Data are shown as means ± SEMs of technical replicates. For, **A-C, E** and **G**, data are representative of two independent experiments. For **B** and **G**, ****P <0.0001,

***P <0.001, **P <0.01, *P<0.05; ns, not significant. P values were calculated by comparing slopes of linear regression.

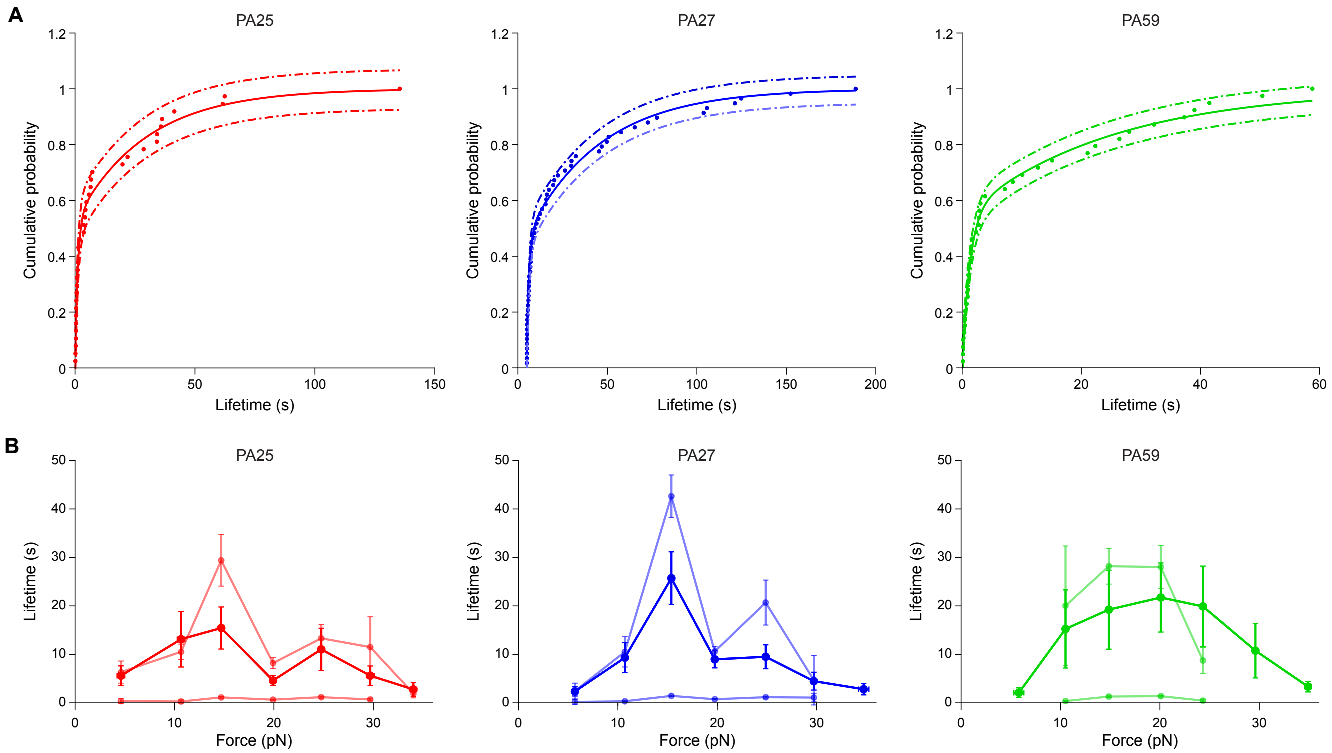
A
PA25
PA27
PA59
Cumulative probability
Lifetime (s)
B
Lifetime (s)
Force (pN)

Fig. S7. Bin by bin cumulative probability distributions fit a double exponential with long- and short- time constants for dissociation.

(A) Double exponential fit to cumulative probability distribution of lifetimes within the 15 pN bin for PA25 (red), PA27 (blue), and PA59 (green). Equation used was $y = A * \left(1 - e^{-x/t1}\right) + B * \left(1 - e^{-x/t2}\right)$. Solid lines show fit with 95% confidence intervals shown by dashed lines. **(B)** Plots comparing time constants from the double exponential fit (light colors) to the averages from the catch bond curves (dark colors) for each clone, respectively. For time constants, 95% confidence for each parameter are shown. In contrast, catch bond averages are plotted with SEM. In each PA receptor system there is an underlying baseline < 2 seconds of quick dissociation events, and a second population of long lifetime events.

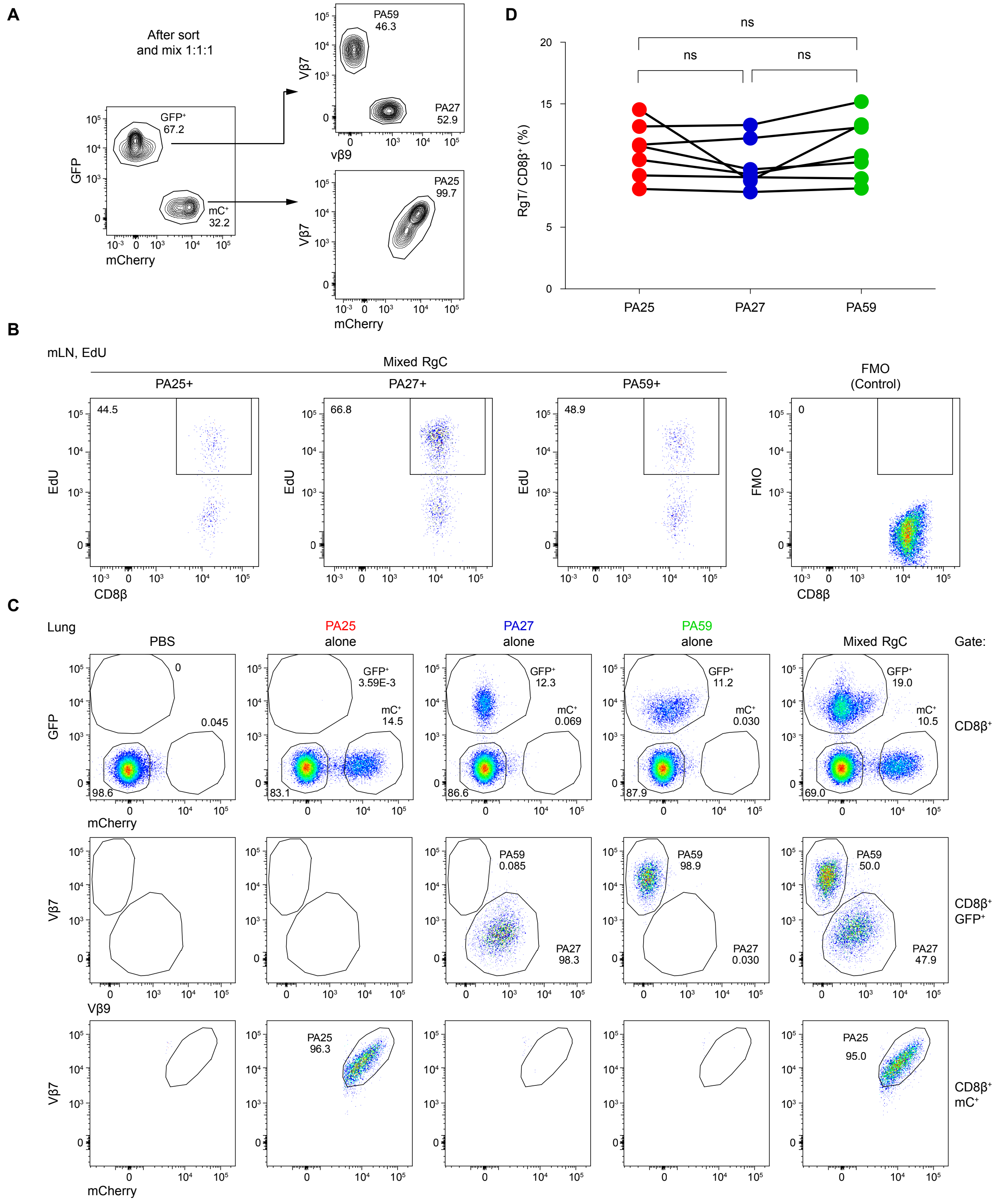
A
After sort and mix 1:1:1
GFP+ 67.2
mC+ 32.2
GFP
mCherry
PA59 46.3
PA27 52.9
Vβ7
vβ9
PA25 99.7
Vβ7
mCherry
D
ns
ns
ns
RgT/ CD8β+ (%)
PA25
PA27
PA59
B
mLN, EdU
Mixed RgC
PA25+
PA27+
PA59+
FMO (Control)
44.5
66.8
48.9
0
EdU
FMO
CD8β
C
Lung
PBS
PA25 alone
PA27 alone
PA59 alone
Mixed RgC
Gate:
GFP+ 3.59E-3
mC+ 14.5
GFP+ 12.3
mC+ 0.069
GFP+ 11.2
mC+ 0.030
GFP+ 19.0
mC+ 10.5
0
0.045
98.6
83.1
86.6
87.9
69.0
CD8β+
GFP
mCherry
PA59 0.085
PA27 98.3
PA59 98.9
PA27 0.030
PA59 50.0
PA27 47.9
CD8β+ GFP+
Vβ7
Vβ9
PA25 96.3
PA25 95.0
CD8β+ mC+
Vβ7
mCherry

Fig. S8. Analysis of PA-specific T cells in mixed RgC mice after IAV infection.

(**A**) Representative FACS plots of RgT cells after cell sorting and mixing PA25, 27, and 59 RgT cells at a 1:1:1 ratio. PA25 is identified as $mCherry^{+}$ $V\beta7^{+}$, PA27 is as GFP^{+} $V\beta9^{+}$, PA59 is as GFP^{+} $V\beta7^{+}$ cells. Those cells are adoptively transferred into recipient B6 mice to generate mixed RgC mice. (**B)** Representative FACS plots of EdU incorporation in PA25, PA27, and PA59 RgT cells in mediastinal LN (mLN) of mixed RgC mice 7 days after PR8 infection, as shown in Fig. 4D. (**C, D)** Representative FACS plots (**C**) of T cell populations in the lungs of mixed RgC as well as individual Rg populations or controls and their quantification (**D**) given as the % of PA25, PA27, and PA59 lung resident Rg T cells of mixed RgC mice 7 days after PR8 infection. For **B-D**, data are representative of four independent experiments. P values were calculated by paired t-test **(D).** ns, not significant.

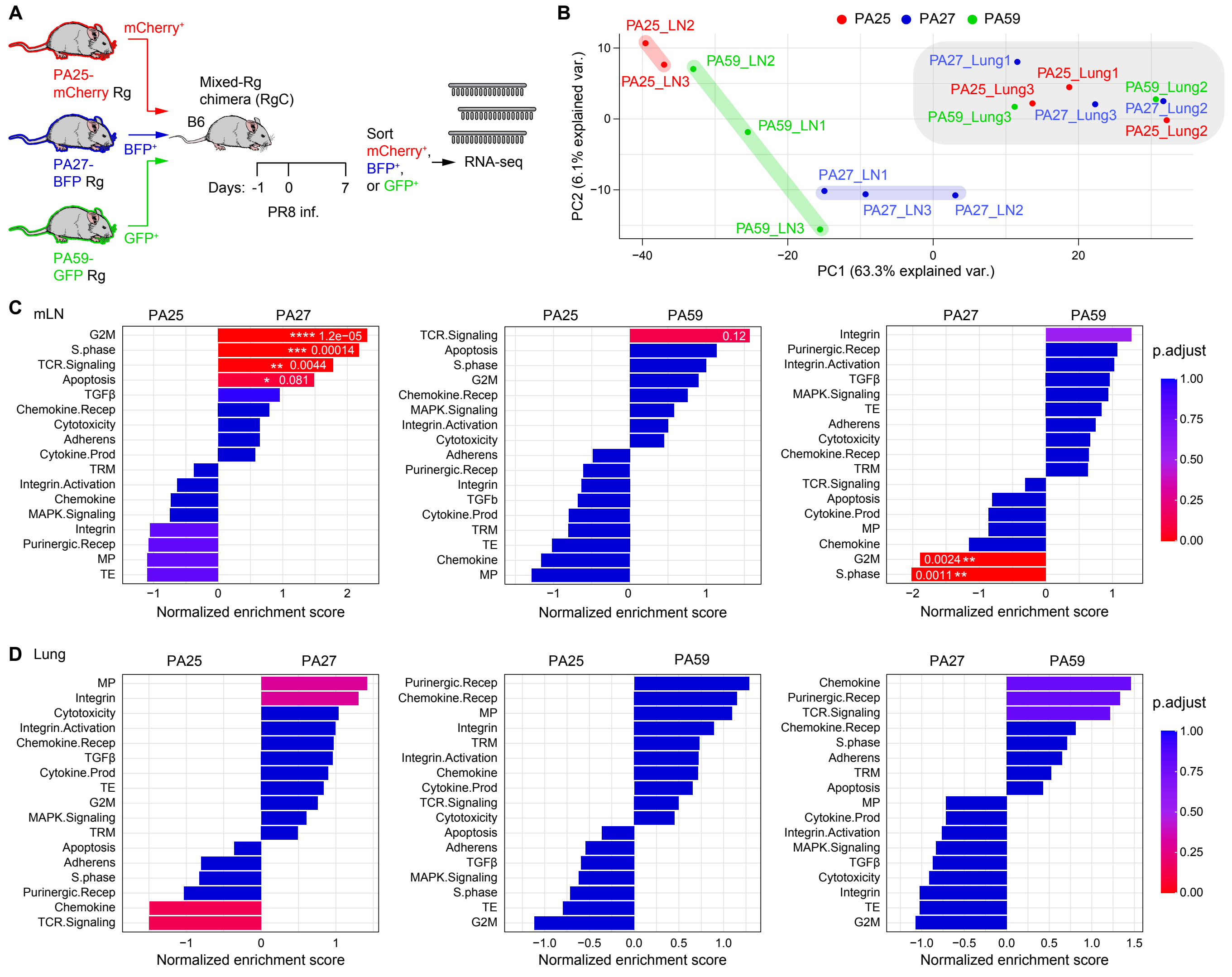

A
mCherry+
PA25-mCherry Rg
BFP+
PA27-BFP Rg
GFP+
PA59-GFP Rg
Mixed-Rg chimera (RgC)
B6
Days: -1 0 7
PR8 inf.
Sort mCherry+, BFP+, or GFP+
RNA-seq
B
PA25 PA27 PA59
PA25_LN2
PA25_LN3
PA59_LN2
PA59_LN1
PA59_LN3
PA27_LN1
PA27_LN3
PA27_LN2
PA27_Lung1
PA25_Lung1
PA25_Lung3
PA59_Lung2
PA27_Lung2
PA59_Lung3
PA27_Lung3
PA25_Lung2
PC1 (63.3% explained var.)
PC2 (6.1% explained var.)
C
mLN
PA25 PA27
**** 1.2e−05
*** 0.00014
** 0.0044
* 0.081
PA25 PA59
0.12
PA27 PA59
0.0024 **
0.0011 **
Normalized enrichment score
p.adjust
D
Lung
PA25 PA27
PA25 PA59
PA27 PA59
Normalized enrichment score
p.adjust

Fig. S9. mLN PA27 T cells are strongly transcriptionally activated after IAV infection.

(**A**) Experimental schema of a TCR labeling and sorting system for bulk RNA-seq. Different fluorescence-tagged and TCR-expressing PA-Rg T cells were sorted as fluorescence$^+$ CD8β$^+$CD44$^-$, mixed with at a ratio of 1:1:1, and then adoptively transferred into recipient B6 mice. These animals then were infected with PR8 24 hours post-transfer. At seven-days post-infection, Rg T cells in mLN or lungs of the mixed RgC mice were sorted as fluorescence protein$^+$, intravenous staining of CD8α$^-$, and *in vitro* staining of CD8β$^+$ cells and populations used to perform RNA-seq. No anti-TCR mAbs were used for this experiment. Gating for cell sorting is shown in data S3. (**B)** Principal Component Analysis (PCA) of mLN and lung samples for PA25-, PA27-, and PA59-Rg T cells. (**C, D)** Gene Set Enrichment Analysis (GSEA) based on pair-wise gene expression comparison between PA-Rg T cell samples in mLN (**C**) and lung (**D**). Statistical significance of gene set enrichment is indicated with asterisks for several thresholds of adjusted P-values (****P < 0.0001, ***P < 0.001, **P < 0.01, *P < 0.1).

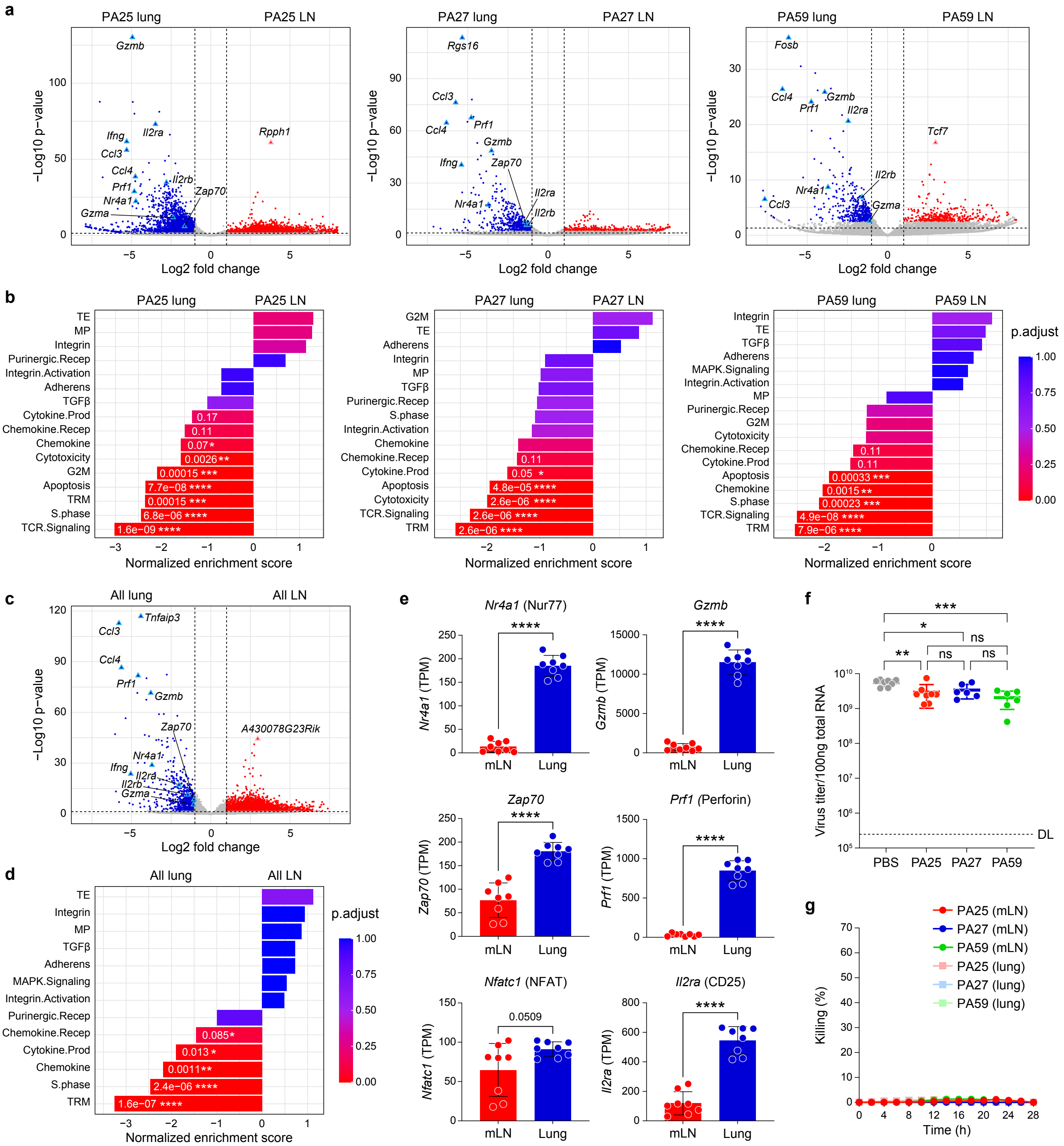

a
PA25 lung
PA25 LN
PA27 lung
PA27 LN
PA59 lung
PA59 LN
−Log10 p−value
Log2 fold change
Gzmb
Ifng
Il2ra
Ccl3
Ccl4
Prf1
Nr4a1
Gzma
Il2rb
Zap70
Rpph1
Rgs16
Fosb
Tcf7
b
PA25 lung
PA25 LN
TE
MP
Integrin
Purinergic.Recep
Integrin.Activation
Adherens
TGFβ
Cytokine.Prod
Chemokine.Recep
Chemokine
Cytotoxicity
G2M
Apoptosis
TRM
S.phase
TCR.Signaling
0.17
0.11
0.07 *
0.0026 **
0.00015 ***
7.7e−08 ****
0.00015 ***
6.8e−06 ****
1.6e−09 ****
PA27 lung
PA27 LN
0.11
0.05 *
4.8e−05 ****
2.6e−06 ****
2.6e−06 ****
2.6e−06 ****
PA59 lung
PA59 LN
MAPK.Signaling
0.11
0.11
0.00033 ***
0.0015 **
0.00023 ***
4.9e−08 ****
7.9e−06 ****
Normalized enrichment score
p.adjust
1.00
0.75
0.50
0.25
0.00
c
All lung
All LN
Tnfaip3
A430078G23Rik
d
All lung
All LN
0.085*
0.013 *
0.0011**
2.4e−06 ****
1.6e−07 ****
e
Nr4a1 (Nur77)
Gzmb
Zap70
Prf1 (Perforin)
Nfatc1 (NFAT)
Il2ra (CD25)
Nr4a1 (TPM)
Gzmb (TPM)
Zap70 (TPM)
Prf1 (TPM)
Nfatc1 (TPM)
Il2ra (TPM)
****
0.0509
mLN
Lung
f
***
*
ns
**
ns
ns
Virus titer/100ng total RNA
DL
PBS
PA25
PA27
PA59
g
PA25 (mLN)
PA27 (mLN)
PA59 (mLN)
PA25 (lung)
PA27 (lung)
PA59 (lung)
Killing (%)
Time (h)

Fig. S10. All lung Rg T cells significantly increase various activation genes after IAV infection.

(**A**) Volcano plots showing differentially expressed genes (DEGs) between mLN and lung of PA25 (left), PA27 (middle), and PA59 (right). Significantly up- and down-regulated genes indicated with red and blue dots (fold-change threshold of 2 and adjusted P-value threshold of 0.05). Remarkably expressing effector-, cytotoxic-, and TCR signaling-genes are labeled and shown as triangles. Note that genes up in the volcano plot on the right side manifest greater expression in mediastinal lymph node relative to those genes in lung on the left and, conversely, those genes on the left side are expressed at a higher level in lung than those in mediastinal lymph node on the right. (**B**) GSEA results based on gene expression comparison between mLN and lung of PA25 (left), PA27 (middle), and PA59 (right). Statistical significance is indicated for several thresholds of adjusted P-values with asterisks (****P < 0.0001, ***P < 0.001, **P < 0.01, *P < 0.1). (**C**,**D**) Volcano and GSEA plots similar to (**A**) and (**B**), but representing results for the comparisons of aggregated Rg T cells in mLN and lung. (**E**) Indicated gene expression in aggregated Rg T cells in mLN and lung. (**F**) Viral titer in lungs of single PA25-, PA27-, PA59-RgC mice (dpi 7) determined by real-time PCR. Control mice were injected PBS without Rg T cells when RgC mice were generated. For **E** and **F**, data are shown as means ± SDs of eight samples (**E**) or six to eight mice (F). ****P <0.0001, ***P <0.001, **P <0.01, *P<0.05; ns, not significant. P values were calculated by unpaired t-test. (**G**) Time-course of T cell-mediated killing of LET1 cells infected with PR8 at a dose of $4x10^8$ EID_5. mLN and lung Rg T cells were derived from RgC mice adoptively transferred with the indicated RgT type (dpi 7).

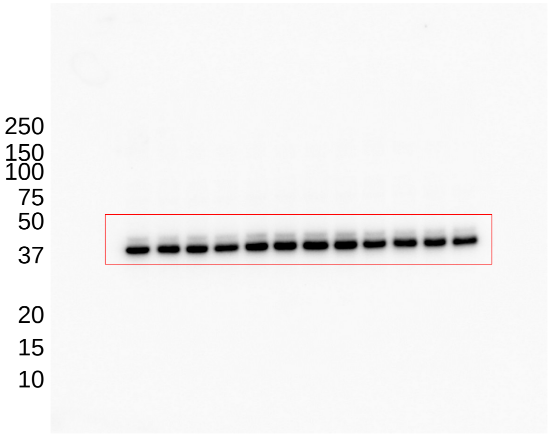

ERK: NP TCR

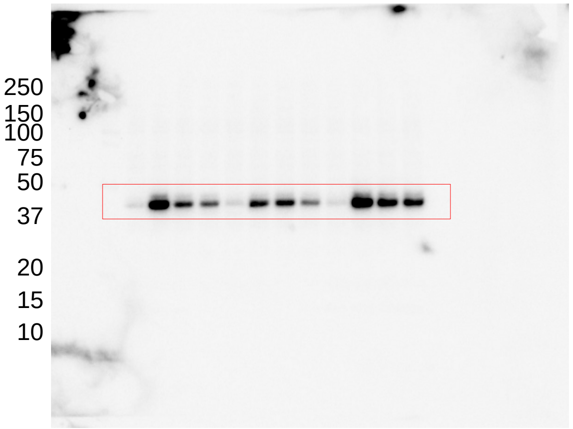

Phosphorylated ERK: NP TCR

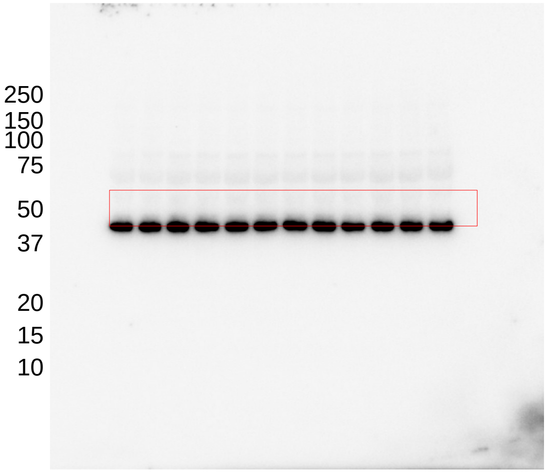

ERK: PA TCR

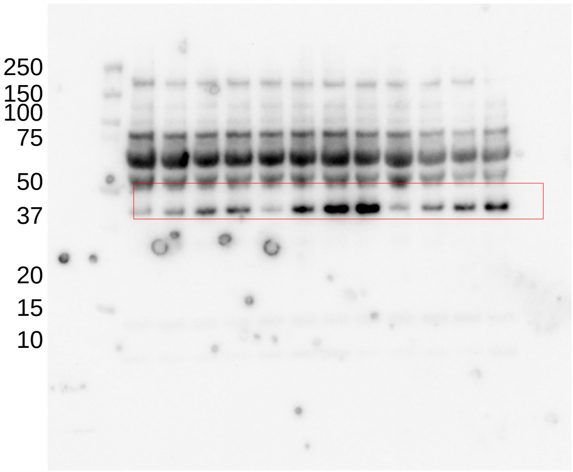

Phosphorylated ERK: PA TCR

Data S2.
Uncropped gels for ERK and pERK expressions of NP- (top) and PA-BW cells (bottom). Related Figs. 1-2.

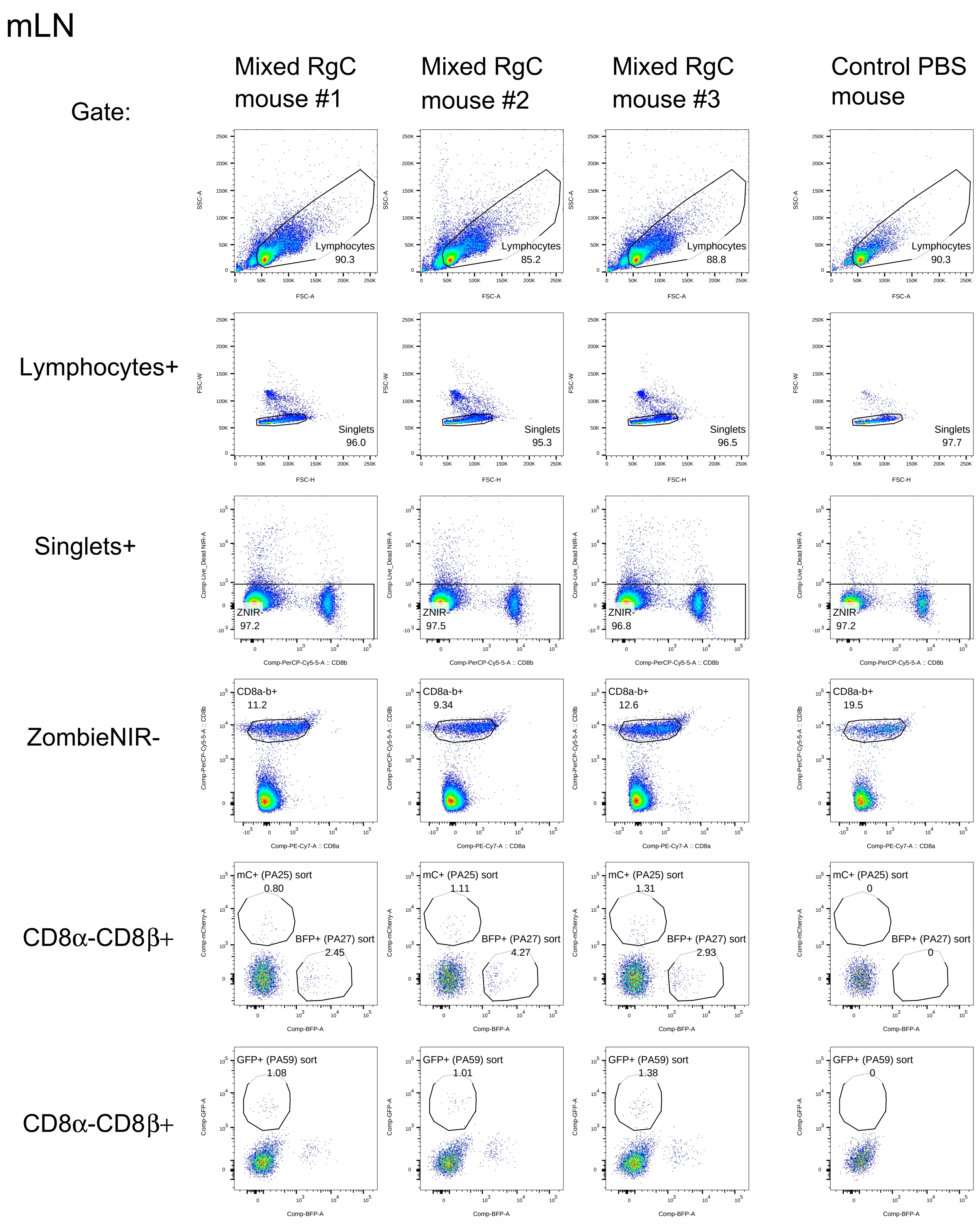
mLN
Gate:
Mixed RgC mouse #1
Mixed RgC mouse #2
Mixed RgC mouse #3
Control PBS mouse
Lymphocytes 90.3
Lymphocytes 85.2
Lymphocytes 88.8
Lymphocytes 90.3
SSC-A
FSC-A
Lymphocytes+
Singlets 96.0
Singlets 95.3
Singlets 96.5
Singlets 97.7
FSC-W
FSC-H
Singlets+
ZNIR- 97.2
ZNIR- 97.5
ZNIR- 96.8
ZNIR- 97.2
Comp-Live_Dead NIR-A
Comp-PerCP-Cy5-5-A :: CD8b
ZombieNIR-
CD8a-b+ 11.2
CD8a-b+ 9.34
CD8a-b+ 12.6
CD8a-b+ 19.5
Comp-PE-Cy7-A :: CD8a
CD8α-CD8β+
mC+ (PA25) sort 0.80
BFP+ (PA27) sort 2.45
mC+ (PA25) sort 1.11
BFP+ (PA27) sort 4.27
mC+ (PA25) sort 1.31
BFP+ (PA27) sort 2.93
mC+ (PA25) sort 0
BFP+ (PA27) sort 0
Comp-mCherry-A
Comp-BFP-A
CD8α-CD8β+
GFP+ (PA59) sort 1.08
GFP+ (PA59) sort 1.01
GFP+ (PA59) sort 1.38
GFP+ (PA59) sort 0
Comp-GFP-A

Lung

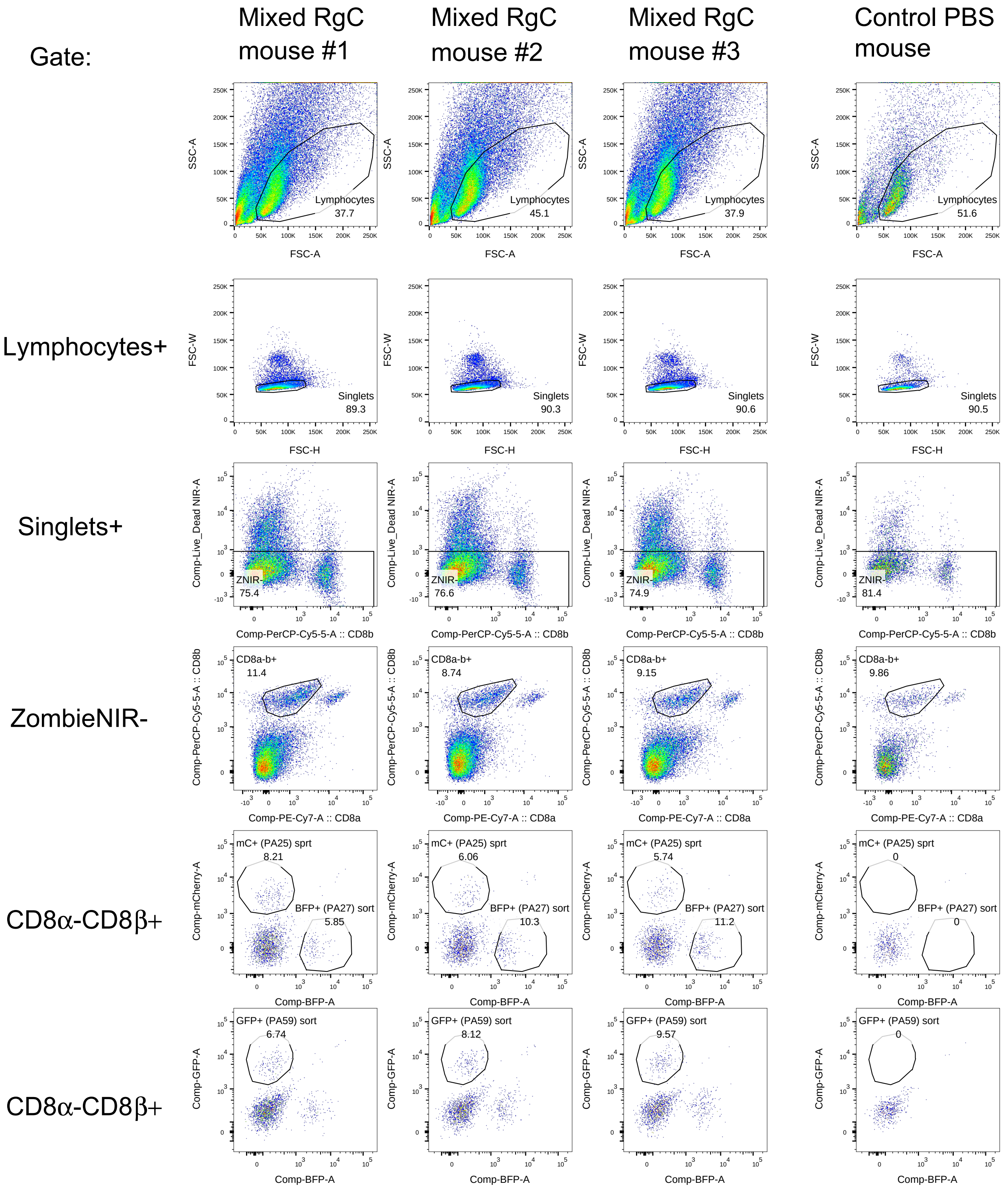
Mixed RgC mouse #1
Mixed RgC mouse #2
Mixed RgC mouse #3
Control PBS mouse
Gate:
SSC-A
FSC-A
Lymphocytes 37.7
Lymphocytes 45.1
Lymphocytes 37.9
Lymphocytes 51.6
Lymphocytes+
FSC-W
FSC-H
Singlets 89.3
Singlets 90.3
Singlets 90.6
Singlets 90.5
Singlets+
Comp-Live_Dead NIR-A
Comp-PerCP-Cy5-5-A :: CD8b
ZNIR- 75.4
ZNIR- 76.6
ZNIR- 74.9
ZNIR- 81.4
ZombieNIR-
Comp-PE-Cy7-A :: CD8a
CD8a-b+ 11.4
CD8a-b+ 8.74
CD8a-b+ 9.15
CD8a-b+ 9.86
CD8α-CD8β+
Comp-mCherry-A
Comp-BFP-A
mC+ (PA25) sprt 8.21
BFP+ (PA27) sort 5.85
mC+ (PA25) sprt 6.06
BFP+ (PA27) sort 10.3
mC+ (PA25) sprt 5.74
BFP+ (PA27) sort 11.2
mC+ (PA25) sprt 0
BFP+ (PA27) sort 0
CD8α-CD8β+
Comp-GFP-A
GFP+ (PA59) sort 6.74
GFP+ (PA59) sort 8.12
GFP+ (PA59) sort 9.57
GFP+ (PA59) sort 0

Data S4. Gating strategy for PA-Rg T cell sorting without utilizing anti-TCR mAbs for RNA-seq.

mLN cells and resident lung cells from 3 mixed RgC mice and control mice.

Name of gene set
Exact name on database
Database_Category_Systematic name
URL
Note

Cell adhesion
GOBP_CELL_CELL_ADHESION
GSEA_C5_M12087
https://www.gsea-msigdb.org/gsea/msigdb/cards/GOBP_CELL_CELL_ADHESION.html
Human HLA genes were excluded from the analysis.

Integrin
REACTOME_INTEGRIN_CELL_SURFACE_INTERACTIONS
GSEA_ C2_ M16441
https://www.gsea-msigdb.org/gsea/msigdb/cards/REACTOME_INTEGRIN_CELL_SURFACE_INTERACTIONS.html

Integrin activation
GOBP_INTEGRIN_ACTIVATION
GSEA_ C5_M23217
https://www.gsea-msigdb.org/gsea/msigdb/cards/GOBP_INTEGRIN_ACTIVATION.html

Cytotoxicity
GOBP_LEUKOCYTE_MEDIATED_CYTOTOXICITY
GSEA_ C5_M11242
https://www.gsea-msigdb.org/gsea/msigdb/cards/GOBP_LEUKOCYTE_MEDIATED_CYTOTOXICITY.html
Human HLA genes were excluded from the analysis.

Cytokine production
GOBP_POSITIVE_REGULATION_OF_T_CELL_CYTOKINE_PRODUCTION
GSEA_ C5_M13565
https://www.gsea-msigdb.org/gsea/msigdb/cards/GOBP_POSITIVE_REGULATION_OF_T_CELL_CYTOKINE_PRODUCTION.html
HLA genes were excluded from the analysis.

Chemokine
GOMF_CCR_CHEMOKINE_RECEPTOR_BINDING
GSEA_ C5_M18725
https://www.gsea-msigdb.org/gsea/msigdb/cards/GOMF_CCR_CHEMOKINE_RECEPTOR_BINDING.html
Cytoplasm genes, JAK1, NARS1, STAT1 were excluded from the analysis.

Chemokine receptor
GOMF_CHEMOKINE_BINDING

GSEA_ C5_M18707
https://www.gsea-msigdb.org/gsea/msigdb/cards/GOMF_CHEMOKINE_BINDING.html
HMGB1 was excluded from the analysis.

Purinergic receptor
GOMF_G_PROTEIN_COUPLED_PURINERGIC_NUCLEOTIDE_RECEPTOR_ACTIVITY
GSEA_ C5_M34434
https://www.gsea-msigdb.org/gsea/msigdb/cards/GOMF_G_PROTEIN_COUPLED_PURINERGIC_NUCLEOTIDE_RECEPTOR_ACTIVITY.html

Apoptosis
KEGG_APOPTOSIS
GSEA_ C2_ M8492
https://www.gsea-msigdb.org/gsea/msigdb/cards/KEGG_APOPTOSIS.html

Adherens_Junction pathway
Adherens junction
KEGG_map04520
https://www.genome.jp/dbget-bin/www_bget?pathway:map04520

TCR signaling pathway
T cell receptor signaling pathway
KEGG_map04660
https://www.genome.jp/dbget-bin/www_bget?pathway:map04660
CD4 was excluded from the analysis.

TGFb signaling pathway
TGF-beta signaling pathway
KEGG_map04350
https://www.genome.jp/dbget-bin/www_bget?pathway:map04350

MAPK signaling pathway
MAPK signaling pathway
KEGG_map04010
https://www.genome.jp/dbget-bin/www_bget?pathway:map04010

Data S8. The sources of gene sets used for Gene Set Enrichment Analysis (GSEA) analysis.

Movie S1.

Ca^{2+} flux of PA25 T cell triggered efficiently under optimal tangential shear force (8-12 pN) but not the other forces (force outside the optimal region). The arrow indicates the direction of the force. The time in the upper left indicates minutes: seconds.

Movie S2.

Ca^{2+} flux of PA27 T cell triggered efficiently under optimal tangential shear force (8-12 pN) but not the other forces (force outside the optimal region). The arrow indicates the direction of the force. The time in the upper left indicates minutes: seconds.

Movie S3.

Ca^{2+} flux of PA59 T cell triggered efficiently under optimal tangential shear force (16-18 pN) but not the other forces (force outside the optimal region). Note that a weak Ca^{2+} flux of PA59 T cell can be triggered under force ranging from 8 to 12 pN. The arrow indicates the direction of the force. The time in the upper left indicates minutes: seconds.

Data S1. (separate file)

TRV, TRJ, and CDR3 sequence for $NP_{366-374}/D^b$- and $PA_{224-233}/D^b$- specific TCRs identified by single-cell RNA-seq.

Data S3. (separate file)

Average fluorescence of pooled single cell calcium flux transients. All curves fit to $y=A*(1-e^{-x/t})$, where y is the fluorescence intensity, A is the amplitude, t is the rise-time constant (s), and x is the time (s). Additionally, the three PA lines include a sine component of $A*\sin(B*x+C)$ added to their curve fits where B and x have units of 1/s and s, respectively. NP34's curve fit starts at t=60s.

Data S5. (separate file)

Gene expression in mLN and lung PA25-, 27-, and 59-Rg T cells after IAV infection (dpi 7) obtained by RNA-seq. The expression level is shown by TPM (Transcript Per Million). The samples filled with gray, PA25_LN_m01 and PA59_Lung_m01 were excluded for further analysis due to their low quality of RNA.

Data S6. (separate file)

Antibodies and tetramers used in this study.

Data S7. (separate file)

The primers and detailed conditions used for single-cell RT-PCR.
