# Supplementary material for: Parsing digital or analog TCR performance through piconewton forces
Source: Sci Adv. 2024 Aug 14;10(33):eado4313. doi: 10.1126/sciadv.ado4313 (PMC11323890; doi:10.1126/sciadv.ado4313)
Supplement: Supplementary file 3 — Data S1 to S8 [file sciadv.ado4313_data_s1_to_s8.zip › ado4313_data_s4.pdf]

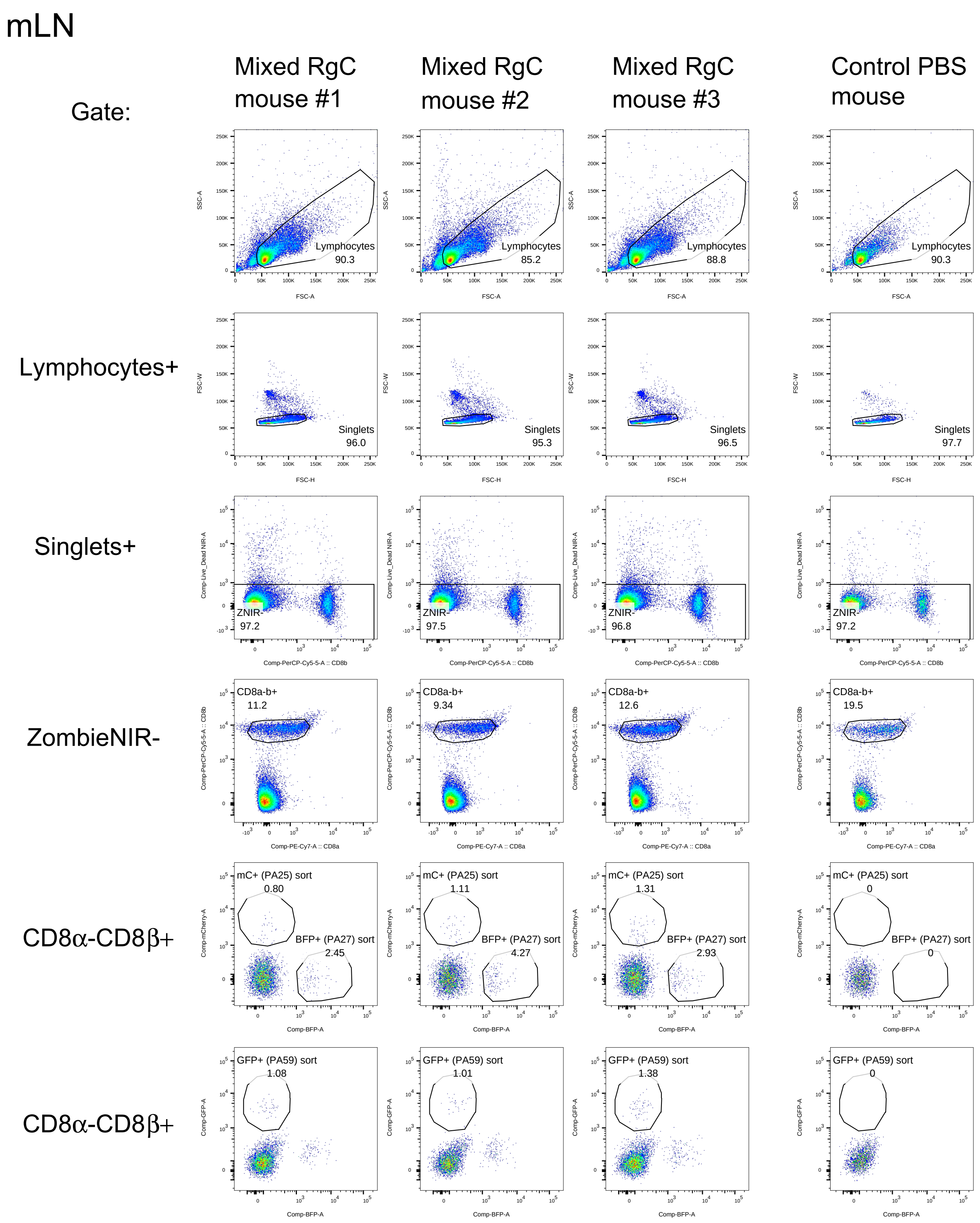
mLN
Gate:
Mixed RgC mouse #1
Mixed RgC mouse #2
Mixed RgC mouse #3
Control PBS mouse
Lymphocytes 90.3
Lymphocytes 85.2
Lymphocytes 88.8
Lymphocytes 90.3
SSC-A
FSC-A
Lymphocytes+
Singlets 96.0
Singlets 95.3
Singlets 96.5
Singlets 97.7
FSC-W
FSC-H
Singlets+
ZNIR- 97.2
ZNIR- 97.5
ZNIR- 96.8
ZNIR- 97.2
Comp-Live_Dead NIR-A
Comp-PerCP-Cy5-5-A :: CD8b
ZombieNIR-
CD8a-b+ 11.2
CD8a-b+ 9.34
CD8a-b+ 12.6
CD8a-b+ 19.5
Comp-PE-Cy7-A :: CD8a
CD8α-CD8β+
mC+ (PA25) sort 0.80
BFP+ (PA27) sort 2.45
mC+ (PA25) sort 1.11
BFP+ (PA27) sort 4.27
mC+ (PA25) sort 1.31
BFP+ (PA27) sort 2.93
mC+ (PA25) sort 0
BFP+ (PA27) sort 0
Comp-mCherry-A
Comp-BFP-A
CD8α-CD8β+
GFP+ (PA59) sort 1.08
GFP+ (PA59) sort 1.01
GFP+ (PA59) sort 1.38
GFP+ (PA59) sort 0
Comp-GFP-A
Comp-BFP-A

Lung

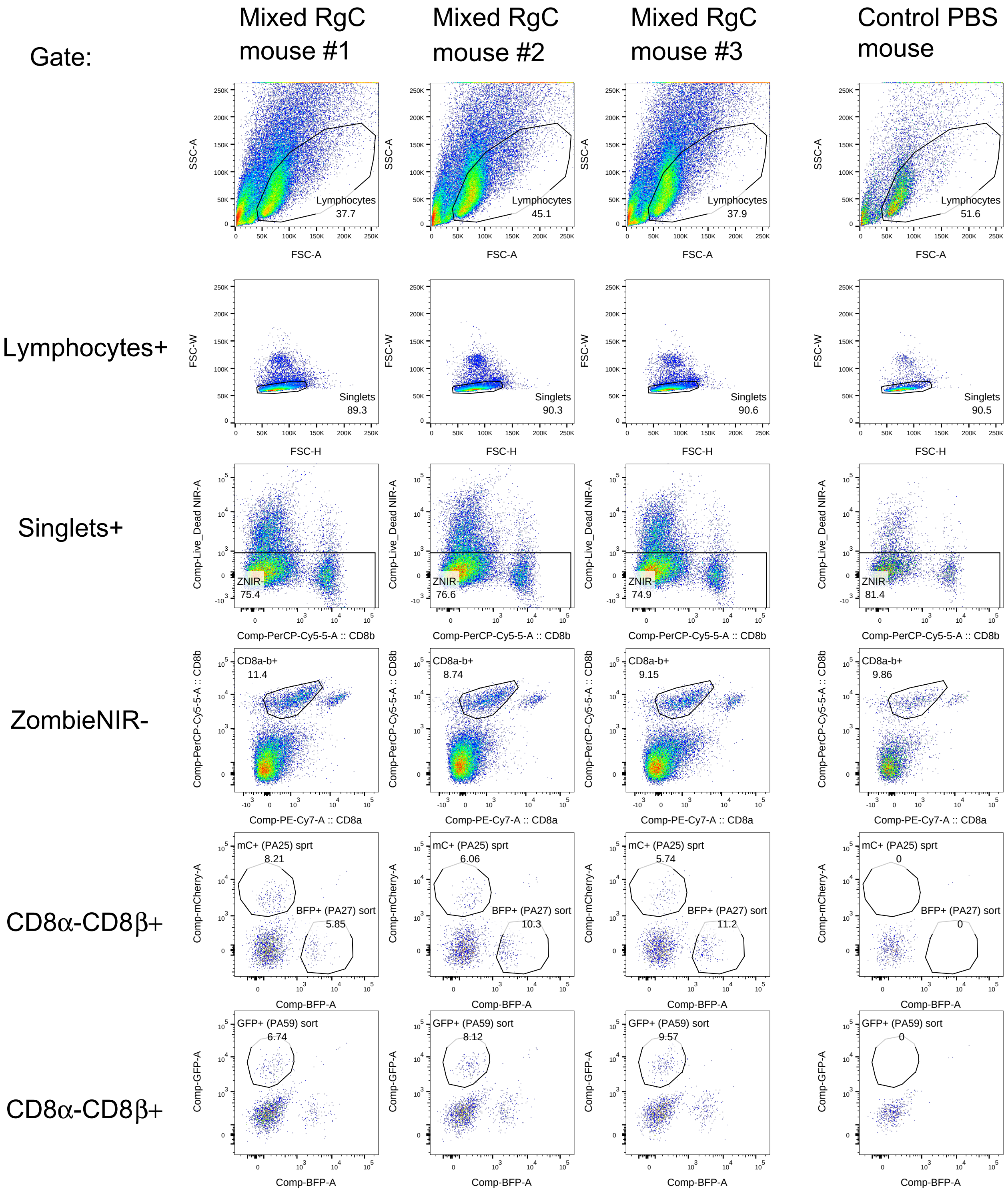

Gate:
Mixed RgC mouse #1
Mixed RgC mouse #2
Mixed RgC mouse #3
Control PBS mouse
SSC-A
FSC-A
Lymphocytes 37.7
Lymphocytes 45.1
Lymphocytes 37.9
Lymphocytes 51.6
Lymphocytes+
FSC-W
FSC-H
Singlets 89.3
Singlets 90.3
Singlets 90.6
Singlets 90.5
Singlets+
Comp-Live_Dead NIR-A
Comp-PerCP-Cy5-5-A :: CD8b
ZNIR- 75.4
ZNIR- 76.6
ZNIR- 74.9
ZNIR- 81.4
ZombieNIR-
Comp-PerCP-Cy5-5-A :: CD8b
Comp-PE-Cy7-A :: CD8a
CD8a-b+ 11.4
CD8a-b+ 8.74
CD8a-b+ 9.15
CD8a-b+ 9.86
CD8α-CD8β+
Comp-mCherry-A
Comp-BFP-A
mC+ (PA25) sprt 8.21
BFP+ (PA27) sort 5.85
mC+ (PA25) sprt 6.06
BFP+ (PA27) sort 10.3
mC+ (PA25) sprt 5.74
BFP+ (PA27) sort 11.2
mC+ (PA25) sprt 0
BFP+ (PA27) sort 0
CD8α-CD8β+
Comp-GFP-A
Comp-BFP-A
GFP+ (PA59) sort 6.74
GFP+ (PA59) sort 8.12
GFP+ (PA59) sort 9.57
GFP+ (PA59) sort 0
