# Supplementary figures and images for: Parsing digital or analog TCR performance through piconewton forces
Source: Sci Adv. 2024 Aug 14;10(33):eado4313. doi: 10.1126/sciadv.ado4313 (PMC11323890; doi:10.1126/sciadv.ado4313)

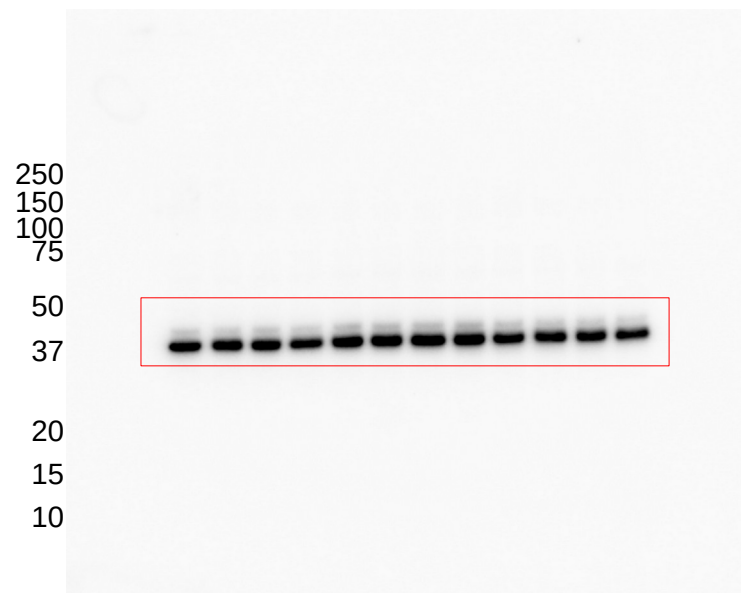

ERK: NP TCR

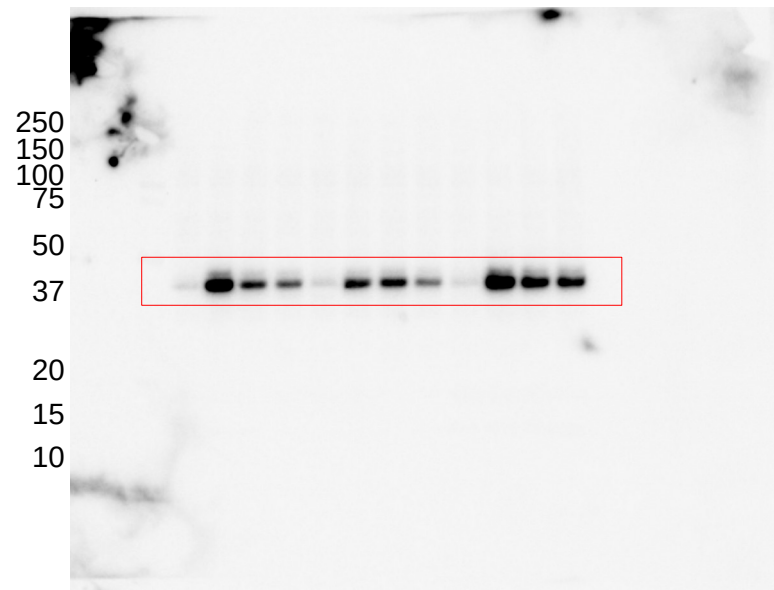

Phosphorylated ERK: NP TCR

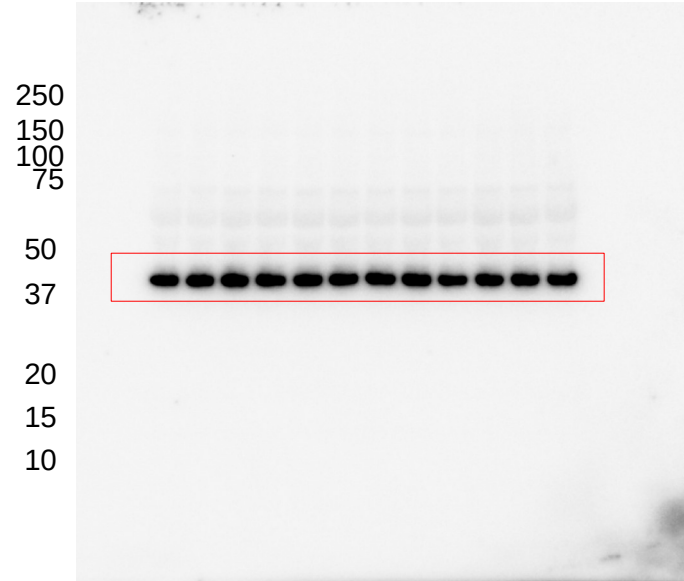

ERK: PA TCR

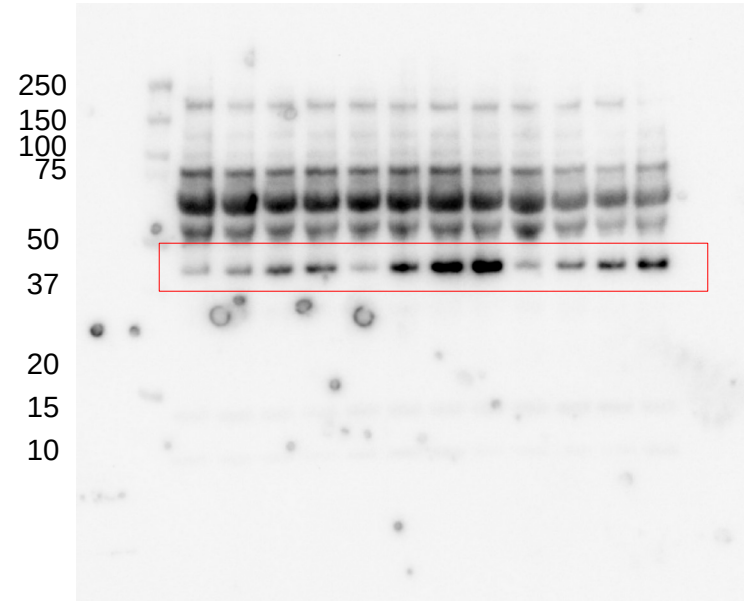

Phosphorylated ERK: PA TCR

Supplement: Supplementary file 3 — Data S1 to S8 [file sciadv.ado4313_data_s1_to_s8.zip › ado4313_data_s2.pdf]
